# 3D Chiral Photonic Nanostructures Based on Blue‐Phase Liquid Crystals

**DOI:** 10.1002/smsc.202100007

**Published:** 2021-05-05

**Authors:** Yanzhao Yang, Ling Wang, Huai Yang, Quan Li

**Affiliations:** ^1^ School of Materials Science and Engineering Tianjin University Tianjin 300350 China; ^2^ Department of Materials Science and Engineering College of Engineering Peking University Beijing 100871 China; ^3^ Institute of Advanced Materials and School of Chemistry and Chemical Engineering Southeast University Nanjing 211189 China; ^4^ Advanced Materials and Liquid Crystal Institute and Chemical Physics Interdisciplinary Program Kent State University Kent OH 44242 USA

**Keywords:** 3D nanostructures, blue-phase liquid crystals, chiral photonics, molecular self-assemblies

## Abstract

3D photonic nanostructures with intrinsic chirality have recently entered the research limelight due to their fundamental importance and potential technological applications. Blue‐phase liquid crystals (BPLCs) with chiral cubic nanostructures are an inventive example of 3D chiral photonic nanostructures. The inherently self‐organized 3D chiral nanostructures give rise to a complete photonic bandgap, which results in the selective reflection of circularly polarized light in all three dimensions. Herein, a comprehensive review of the state‐of‐the‐art of BPLCs and their potential applications is presented. First, the history and fundamentals of BPLCs are introduced. Then, the recent endeavors in the design, synthesis, and properties of BPLCs such as lattice orientation control with different techniques, photonic bandgap tuning with external fields, and fabrication of free‐standing BPLC polymer films are summarized. Finally, a discussion of the future challenges and potential applications of BPLCs is provided. It is believed that this review would stimulate innovative ideas for the design and engineering of novel chiral nanostructured materials for advanced photonic systems with tailorable functionalities.

## Graphical abstract

3D chiral photonic nanostructures are of great significance due to the presence of a complete photonic bandgap and omnidirectional selective reflection of circularly polarized light. Herein, an account on the state‐of‐the‐art in blue‐phase liquid crystals with chiral cubic nanostructures and their potential applications is provided. In addition, perspectives for future scope, challenges, and opportunities for such a multidisciplinary topic are presented.

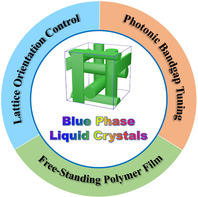

## 1 Introduction

**Figure 1:**
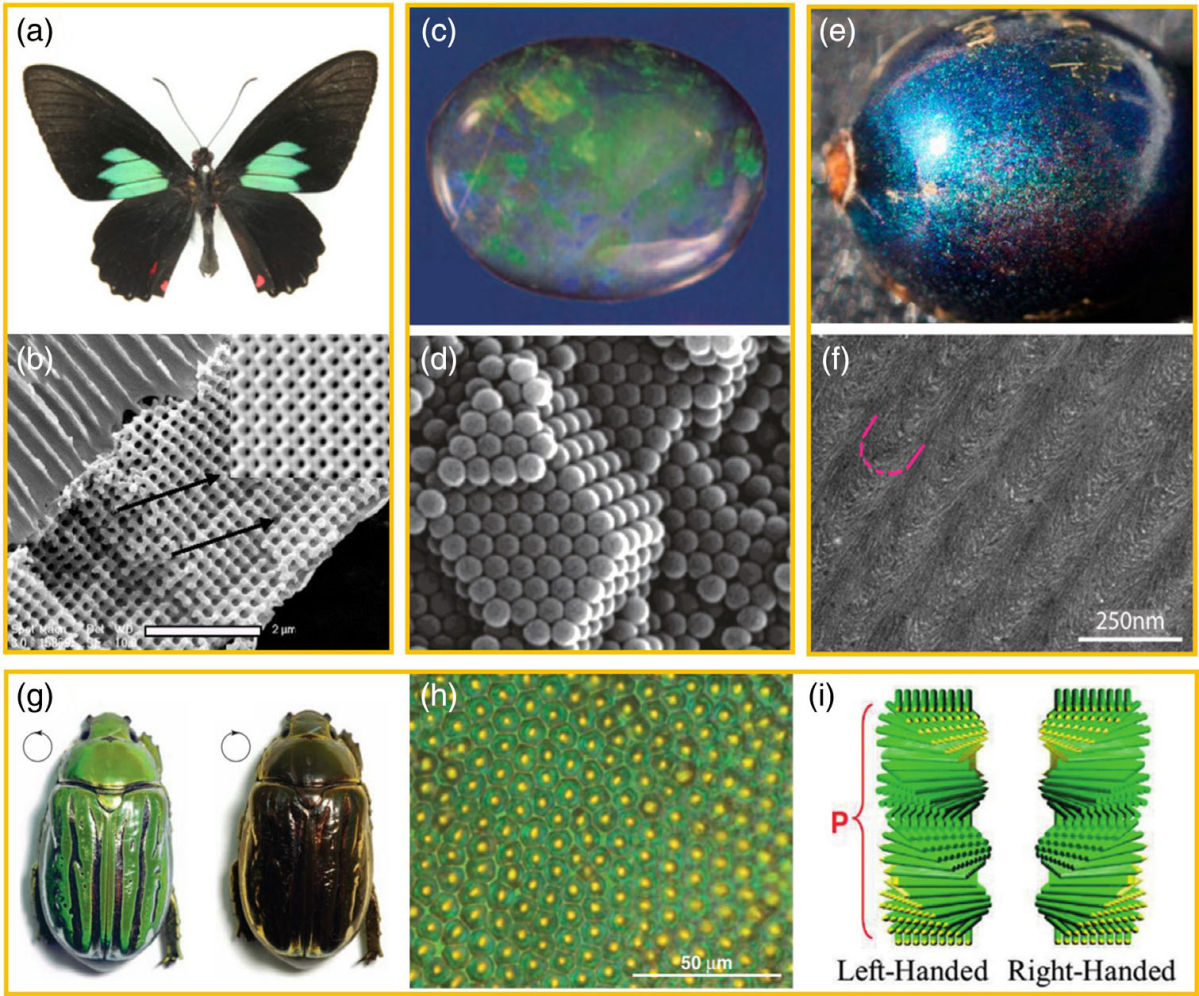
Photonic nanostructures in nature. a,b) Photograph and scanning electron microscopy (SEM) image of the *P. sesostris* butterfly. a) Reproduced with permission.^[^ ^14a^ ^]^ Copyright 2014, The Royal Society. b) Reproduced with permission.^[^ ^14b^ ^]^ Copyright 2010, National Academy of Sciences. c,d) Photograph and SEM image of opal. Reproduced with permission.^[^ ^15^ ^]^ Copyright 2009, Wiley‐VCH. e,f) Photograph and TEM image of *P. condensata* fruits. Reproduced with permission.^[^ ^23^ ^]^ Copyright 2012, National Academy of Sciences. g,h) Appearance and optical micrograph of jeweled beetle *C. gloriosa*. The bright green iridescent color is observed when seen under a left‐circular polarizer and disappears when seen under a right‐circular polarizer. Reproduced with permission.^[^ ^25^ ^]^ Copyright 2009, American Association for the Advancement of Science. i) Schematics of left‐handed and right‐handed organization in chiral helicoidal superstructures. Reproduced with permission.^[^ ^26^ ^]^ Copyright 2014, Elsevier.

Photonic crystals are periodic dielectric nanostructures that exhibit brilliant structural colors by reflecting light of certain wavelengths through multiple light–matter interference. Recently, photonic crystals have attracted significant attention due to their fundamental significance and technological applications in diverse fields such as materials science, optical devices, communication technologies, and biomimetic and biomedical systems.^[^ 1, 2, 3, 4, 5 ^]^ These distinctive well‐defined ordered nanostructures are characterized by the unique photonic bandgap capable of modulating light–matter interactions in specific spectral regions. Photonic bandgaps prevent the propagation of light of wavelengths or frequencies corresponding to the photonic bandgap within the periodic nanostructured materials. Over millions of years of evolution, nature has granted us numerous examples of photonic nanostructures in both terrestrial and aquatic ecosystems.^[^ 6, 7, 8, 9, 10, 11, 12, 13 ^]^ In plants, iridescent colors originating from periodic nanostructures make them more attractive to birds and insects, while iridescence in animals is believed to play a critical role in sexual selection, adaptive camouflage, and warning predators. For example, the brilliant iridescent colors of some butterflies, opals, insects, peacocks, and chameleons are generated by the periodic nanostructures on their surface that reflect light in specific spectral ranges (**Figure** **1**a–d).^[^ 14, 15, 16, 17, 18, 19, 20, 21, 22 ^]^ Many types of periodic photonic nanostructures have been discovered in nature, from 1D multilayered thin films and gratings to 2D nanostructured photonic microarrays and microcavities to 3D colloidal crystals and porous geometries.

Chiral photonic nanostructures can also be found in many living and nonliving systems such as in the cuticles of beetles, arthropods, crabs, and some plant tissues.^[^ 23, 24, 25, 26, 27 ^]^ Chirality is of particular interest in the study of reflection of light by periodic nanostructures, as light itself has a certain degree of chirality. One of the main properties of light in free space, in addition to color, is its polarization state: unlike linearly polarized light, right‐handed and left‐handed circularly polarized lights are chiral. For example, chiral photonic nanostructures have been observed in some plants, such as the *Pollia condensata* fruits (Figure 1e,f). The color is caused by Bragg reflection of helicoidally stacked cellulose microfibrils that form multilayers in the cell walls of the epicarp. Because the multilayers form with both helicoidicities, the reflected light from every epidermal cell is polarized circularly either to the left or to the right.^[^ ^23^ ^]^ Interestingly, the beetle *Chrysina gloriosa* was found to exhibit a bright‐green metallic color and selectively reflects left‐handed circularly polarized light (Figure 1g–i).^[^ 25, 26, 27 ^]^ The beetle's surface appears iridescent vivid green with black stripes when observed under a left‐circular polarizer or with unpolarized light; however, the green color mostly disappears when observed under a right‐circular polarizer. Circular polarization plays a very important role in our daily life although the human eye cannot resolve it. Modern 3D cinema technology is known to result from two slightly shifted images of opposite circular polarizations, and 3D effect is enabled since each polarization can only be seen through one eye if the corresponding glasses are worn. This kind of 3D glasses share the same mechanism as that demonstrated by the beetle *C. gloriosa*, which is considered as one of the most striking examples of circular polarization‐sensitive reflection in the animal kingdom.

Biomimetic iridescent photonic nanostructures can be traced back to 1987, when Yablonovitch and John fabricated artificial photonic crystals inspired by nature.^[^ 28, 29 ^]^ To date, photonic nanostructures are generally fabricated via two approaches: top‐down and bottom‐up. The top‐down approach is mostly based on conventional microfabrication techniques such as etching and photolithography to produce microstructures with desired shapes, orders, and sizes from bulk materials.^[^ 30, 31, 32 ^]^ The bottom‐up approach, however, involves the controlled self‐assembly of judiciously designed building blocks into periodic photonic nanostructures.^[^ 33, 34, 35, 36, 37 ^]^ Although significant advancements have been made in the development of various artificial photonic nanostructures, the fabrication of 3D nanostructures with intrinsic chirality remains a challenging task. Based on the theoretical prediction of large complete 3D photonic bandgaps in high‐index contrast silicon square‐spiral structures,^[^ 38, 39 ^]^ Thiel et al. fabricated 3D chiral photonic crystals by a two‐photon direct laser writing technique; the high‐quality 3D polymeric helices arranged in a periodic array exhibited polarization stop bands with large circular dichroism in transmission in the infrared regions.^[^ ^40^ ^]^ They also prepared more complicated bichiral dielectric photonic crystals with cubic symmetry and pronounced polarization stop bands.^[^ ^41^ ^]^ The resulting 3D “bichiral” photonic nanostructures simultaneously exhibited two distinct types of chirality, where one type of handedness originated from the circular spirals arranged on a simple cubic 3D lattice, and a second type of chirality was introduced by the orientation of the three ﬁctitious spiral axes. Furthermore, inspired by the chiral light‐generating nanostructures in the wings of *Callophrys rubi* butterfly, complete circular polarization stop bands have been recently demonstrated in gyroid photonic crystals.^[^ 42, 43 ^]^ It should be noted that undesirable defects or dislocations often inevitably generate in traditional bottom‐up fabrication processes involving controlled self‐assembly, whereas lattice distortion or shrinkage often occurs in top‐down fabrication processes.

Liquid crystals combine the fluidity of conventional liquids and the ordering of crystals from molecular to macroscopic length scales and are considered one of the most promising materials for the development of large‐scale, high‐quality chiral photonic crystals.^[^ 44, 45, 46, 47, 48, 49, 50, 51, 52, 53, 54, 55 ^]^ Chiral liquid‐crystalline materials with inherent self‐organization ability are a promising candidate for the efficient, “green” and low‐cost production of photonic nanostructures with circular dichroism. The “softness” of liquid‐crystalline materials with tunable photonic bandgaps and robust responsiveness render them sensitive to various stimuli such as light, temperature, electric field, mechanical force, and chemical and electrochemical reactions.^[^ 56, 57, 58, 59, 60, 61, 62, 63 ^]^ In this regard, 1D photonic superstructures based on cholesteric liquid crystals (CLCs) have been extensively investigated and widely applied in reflective displays, tunable lasers, and other advanced chiral photonic devices.^[^ 64, 65, 66, 67, 68, 69, 70, 71, 72, 73, 74, 75, 76, 77, 78, 79 ^]^ Blue‐phase liquid crystals (BPLCs) with cubic lattice nanostructures have recently entered the research limelight due to their ability to self‐organize into 3D chiral photonic crystals that can manipulate light or the flow of photons in all the three dimensions.^[^ 80, 81, 82, 83, 84, 85 ^]^ Herein, we present a comprehensive review of the state‐of‐the‐art in 3D chiral photonic nanostructures based on BPLCs and their potential applications. We first present the fundamentals of 3D chiral architectures in BPLCs, and then discuss the recent endeavors in the development of BPLCs, including lattice orientation control with different techniques, photonic bandgap tuning with external fields, and fabrication of free‐standing blue phase (BP) polymer films. We conclude the review with a discussion on the future challenges and opportunities for these emerging 3D chiral photonic nanostructures and their potential applications. We believe that this Review would provide a deeper understanding of BPLCs and stimulate innovative ideas for the design and engineering of new chiral nanostructured materials for advanced photonic systems with tailorable properties and novel functionalities.

## 2 Fundamentals of BPLCs

**Figure 2:**
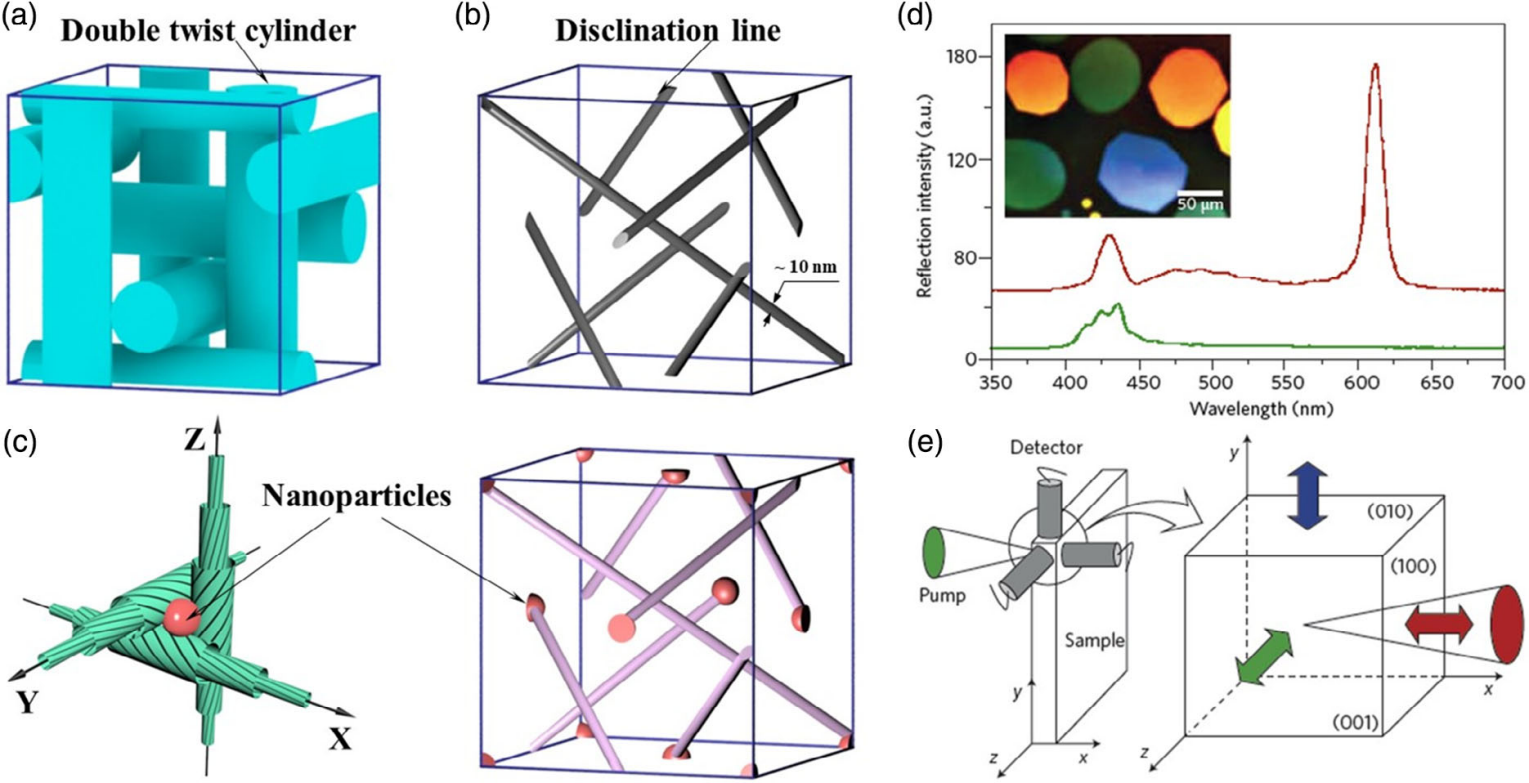
a) Schematic arrangement of double twist cylinders in a body‐centered cubic nanostructure. b) 3D disclination network in BP I. a,b) Reproduced with permission.^[^ ^111^ ^]^ Copyright 2013, Royal Society of Chemistry. c) Schematic illustration of nanoparticle‐stabilized BP nanostructures.^[^ ^108^ ^]^ Copyright 2012, Wiley‐VCH. d) Optical texture and reflection spectra of BP single crystals (red) and cholesteric liquid crystals (green). e) Schematic illustration of laser emissions from BP nanostructures in three orthogonal directions. d,e) Reproduced with permission.^[^ ^95^ ^]^ Copyright 2002, Springer Nature.

When chirality is introduced into achiral nematic liquid crystals, the adjacent mesogenic molecules slightly twist with each other to generate a chiral nematic phase with a helical superstructure.^[^ ^56^ ^]^ In this case, the twisted molecular structure is formed along a single axis in the lateral molecular direction (perpendicular to the long axis), which is known as a “simple twist.” However, no twisting is induced in the directions that perpendicular to the helical axis. If the liquid‐crystal molecules can be twisted simultaneously in both directions, the mesogenic molecules tend to self‐organize into a “double‐twist” molecular arrangement, which is known to form the double‐twisted cylinders (DTCs). As the DTCs are unable to occupy a space continuously, the defects inevitably occur in such molecular arrangements. This is the reason why the simple twist is common structure in nature rather than the double twist, and BPLCs are built upon such a DTC structure that maintains this inconsistency.^[^ 80, 85 ^]^ The name “blue phase” (BP) derives from the blue color of the liquid‐crystalline phase when it was first discovered.^[^ ^86^ ^]^ Depending on their chirality upon cooling from the isotropic phase to cholesteric phase, BPs can be classified as BP III, BP II, and BP I. BP III is often considered a disordered amorphous nanostructure,^[^ 87, 88 ^]^ whereas BP II has a simple cubic symmetry and BP I has a body‐centered cubic nanostructure. Because the DTCs are unable to continuously fill the entire 3D space, defects, or disclinations unavoidably form in BP nanostructures (**Figure** **2**a–c).

With a submicrometer‐scale lattice spacing, BPLCs usually exhibit selective photonic bandgaps in the visible range. Therefore, BPLCs are considered one of the most promising candidates for the highly efficient production of 3D photonic crystals.^[^ 89, 90, 91, 92, 93 ^]^ According to Bragg's law, the reflective wavelength (*λ*) of BP nanostructures can be determined by the following equation^[^ ^94^ ^]^

(1) $$\lambda =\frac{2na\cos\theta }{\sqrt{h^{2}+k^{2}+l^{2}}}$$

where *n* represents the average refractive index, *a* represents the lattice constant of the BPLCs, *h*, *k*, and *l* are the Miller indices of crystal orientation planes, and *θ* is the angle between the crystallographic direction [*h, k, l*] and incident light. Due to their inherent liquid‐crystal properties and 3D‐ordered nanostructure, BP II and BP I exhibit fast response and ultrahigh tunability to various external stimuli such as heat, light, and electric field, which are beneficial for the fabrication of BP‐based optical devices with tunable properties. One of the major advantages of BPLCs is that laser emission in three orthogonal directions can be simultaneously achieved because of the 3D photonic bandgap. This was first reported for BP II cubic nanostructures by Cao et al.,^[^ ^95^ ^]^ who observed sharp peaks at the low‐energy edge of the bandgap in three orthogonal directions (Figure 2d,e). When one of the platelet BP domains in a liquid crystal thin cell is excited by the pump laser beam, laser emissions from distributed feedback in the orthogonal (100), (010), and (001) directions can be observed at the same time, which is actually a sign of the distributed feedback in three dimensions. Compared with the chiral nematic or cholesteric liquid‐crystalline systems, the threshold energy of laser excitation in BPLCs is much lower, and these elegant soft 3D photonic materials could find promising applications in diverse fields such as spatial phase modulators, microring resonators, waveguiding, lenses, gratings, displays, and many other photonic devices.

In general, BPs exist only in a narrow temperature range (≈2 °C), which is one of the most critical issues that limits their potential applications. It is agreed that the high‐energy cost related to the disclinations usually decreases the thermal stability of BPLCs. Thus, the BP temperature range can be extended by filling the space of disclinations with specific materials such as polymers,^[^ 96, 97, 98 ^]^ low‐weight molecules,^[^ 99, 100, 101, 102, 103, 104, 105, 106 ^]^ and inorganic nanoparticles.^[^ 107, 108, 109, 110, 111 ^]^ The stabilization of BPLCs with polymers is the most successful BP stabilization method. Polymer‐stabilized blue phases (PSBPs) were first reported by Kikuchi et al. in 2002.^[^ ^96^ ^]^ The 3D BP nanostructures with self‐organized DTCs were stabilized over a wide temperature range of more than 60 °C by selectively concentrating the polymer network in the disclination lines. In recent years, free‐standing BP polymer films with promising optical performances have been fabricated using reactive mesogens. In these materials, the helical liquid‐crystalline arrangement and the 3D periodic nanostructure are frozen in the polymeric networks.^[^ 112, 113 ^]^ These BP polymer films with superior optical properties could overcome the restriction of liquid‐crystal cells, significantly broadening the application scope of BP nanostructures.

Compared with other chiral photonic nanostructures, BPLCs offer outstanding advantages such as narrow photonic bandgaps, unique 3D periodic nanostructures, and obviating the need for molecular alignment. In addition, their lattice orientation, selective reflection, birefringence, and crystal structure can be easily modulated. Because of their outstanding properties and potential applications in diverse fields, the utilization of BPLCs in electro‐optical devices, information display, photonic materials, and others have been extensively reviewed.^[^ 81, 82, 114, 115, 116 ^]^ Herein, we focus on the fabrication of BPLCs into ideal superstructures, stimuli‐driven 3D chiral BP photonic nanostructures with tailorable photonic bandgaps, and free‐standing BP polymer films for unprecedented applications.

## 3 Lattice Orientation Control of BPLCs

BPLCs usually contain multiple platelet domains because of random nucleation during self‐assembly. The crystalline platelets generally have an average size of ≈10 μm in lateral dimensions. Small domains with random crystallographic orientations and planes tend to increase scattering loss, which decreases the quality factor; hence, multidomain BPLCs usually exhibit a low Bragg reflectance. Compared with traditional multidomain BPLCs, monodomain or monocrystalline BPLCs exhibit many advantageous characteristics, including high reflectivity, reduced hysteresis, and superior electro‐optical properties.^[^ 117, 118, 119 ^]^ Monodomain BPLCs are cubic lattices with a uniform crystallographic plane orientation. It should be noted that grain boundaries still exist between the platelets and interfere with the performance of monodomain BPLCs. A further reduction in grain boundaries would yield monocrystalline BPLCs. Hence, controlling the BP lattice orientations to obtain sufficiently large‐scale monodomain BPLCs or monocrystalline BPLCs for advanced photonic applications is an appealing goal.

### 3.1 Lattice Orientation by Alignment Layers

Surface anchoring force can play a critical role in the molecular orientation of liquid‐crystalline systems. Monodomain BPLCs with a uniform lattice orientation can be achieved using alignment layers.^[^ 120, 121, 122, 123 ^]^ Jiang et al. conducted a comparative study of the optical properties of BPLCs with rubbing alignment layers and photoalignment layers.^[^ ^120^ ^]^ The polarized optical microscope (POM) images of the rubbing‐aligned and photoaligned samples revealed the presence of monodomain BP textures. Moreover, the POM images of the two cells revealed the formation of a more uniform BP texture in the photoalignment layers than that in the rubbing alignment layers. Similarly, Takahashi et al. studied the crystal orientation of BPLCs on two parallelly aligned surfaces obtained by photoalignment and rubbing.^[^ ^121^ ^]^ Upon cooling the samples from the isotropic phase to BP II, different crystal orientations were observed in the two alignment layers: (100) crystal planes were oriented in the photoalignment cell, whereas (110) crystal planes were oriented in the rubbing cell. This indicates that the properties of the alignment layer are as important as the thermal process for the lattice orientation control of BPLCs.

**Figure 3:**
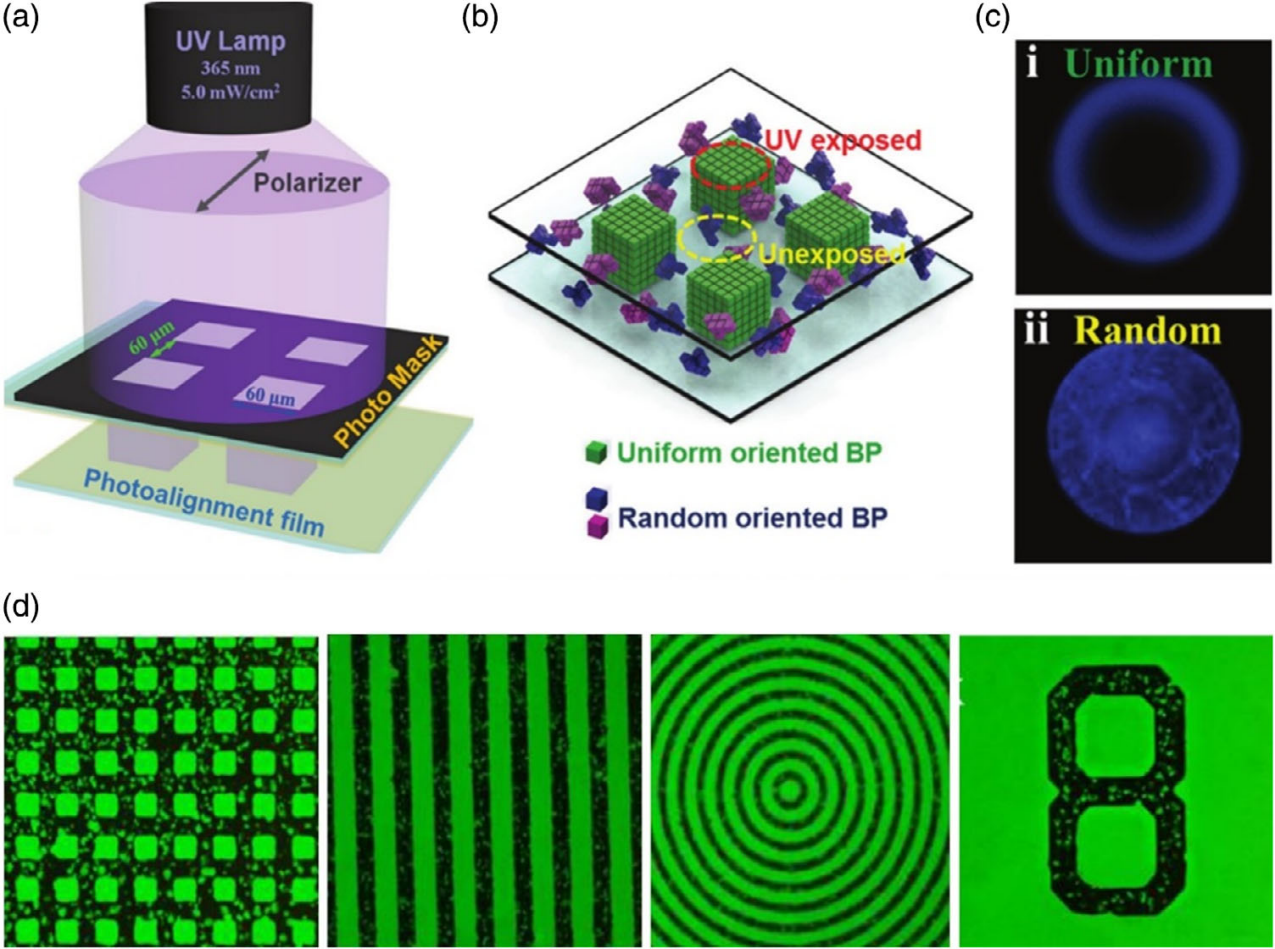
Light‐directed patterned lattice orientation in soft BP specimens. a) Fabrication scheme for light‐directed patterning. b) Schematic of lattice orientation in UV‐exposed regions and unexposed regions. c) Kossel diagrams of uniformly oriented regions and randomly oriented regions. d) POM images of light‐directed patterns. a–d) Reproduced with permission.^[^ ^124^ ^]^ Copyright 2017, Wiley‐VCH.

Light‐directed uniform and patterned orientation of ordered materials is of great interest and has fundamental significance for photonic applications.^[^ 124, 125, 126 ^]^ Zheng et al. proposed the light‐directed patterning of 3D chiral photonic BP nanostructures by regionally selective lattice orientation using photoalignment layers and photomasks (**Figure** **3**a).^[^ ^124^ ^]^ The BP specimens exhibited a uniform lattice orientation in the polarized UV‐exposed areas because of the presence of a photoalignment layer, whereas a random BP lattice orientation was observed in the unexposed areas (Figure 3b). With the help of polarized UV light and a patterned photomask, various patterns with brilliant‐colored and dark regions, corresponding to uniformly oriented BP lattice and randomly oriented BP lattice regions, respectively, were produced. The Kossel diagram of the region with a uniform lattice orientation exhibited a circular ring, whereas that of the unexposed regions exhibited an indistinct pattern with many distorted overlapping lines because of random BP lattice orientation (Figure 3c). Because of the softness of BPLCs, arbitrary patterns could be written and erased by the realignment of BP lattices (Figure 3d). This work provided a new insight into the manipulation of BP lattice orientation for rewriteable displays and functional soft materials.

Although monodomain BP nanostructures have been achieved by the unidirectional alignment of layers, they are polycrystalline structures with small crystalline domains oriented along the alignment axis. To obtain monocrystalline BPLCs, the cubic lattices should be 3D‐oriented in a large scale and the grain boundaries should be completely eliminated. Otón et al. prepared large monocrystalline BPLCs with ideal lattice orientation by a simple surface treatment and controlling the amount of precursor materials.^[^ 127, 128 ^]^ Instead of using traditional polyimide as alignment layers, they used Nylon 6 and Nylon 6‐6 polyamide layers. The anchoring energies of polyamide are one or two orders of magnitude lower than that of polyimides. The weak anchoring energy of the Nylon layers was found to be main factor in the production of monocrystalline BPLCs with superior electro‐optical properties.

**Figure 4:**
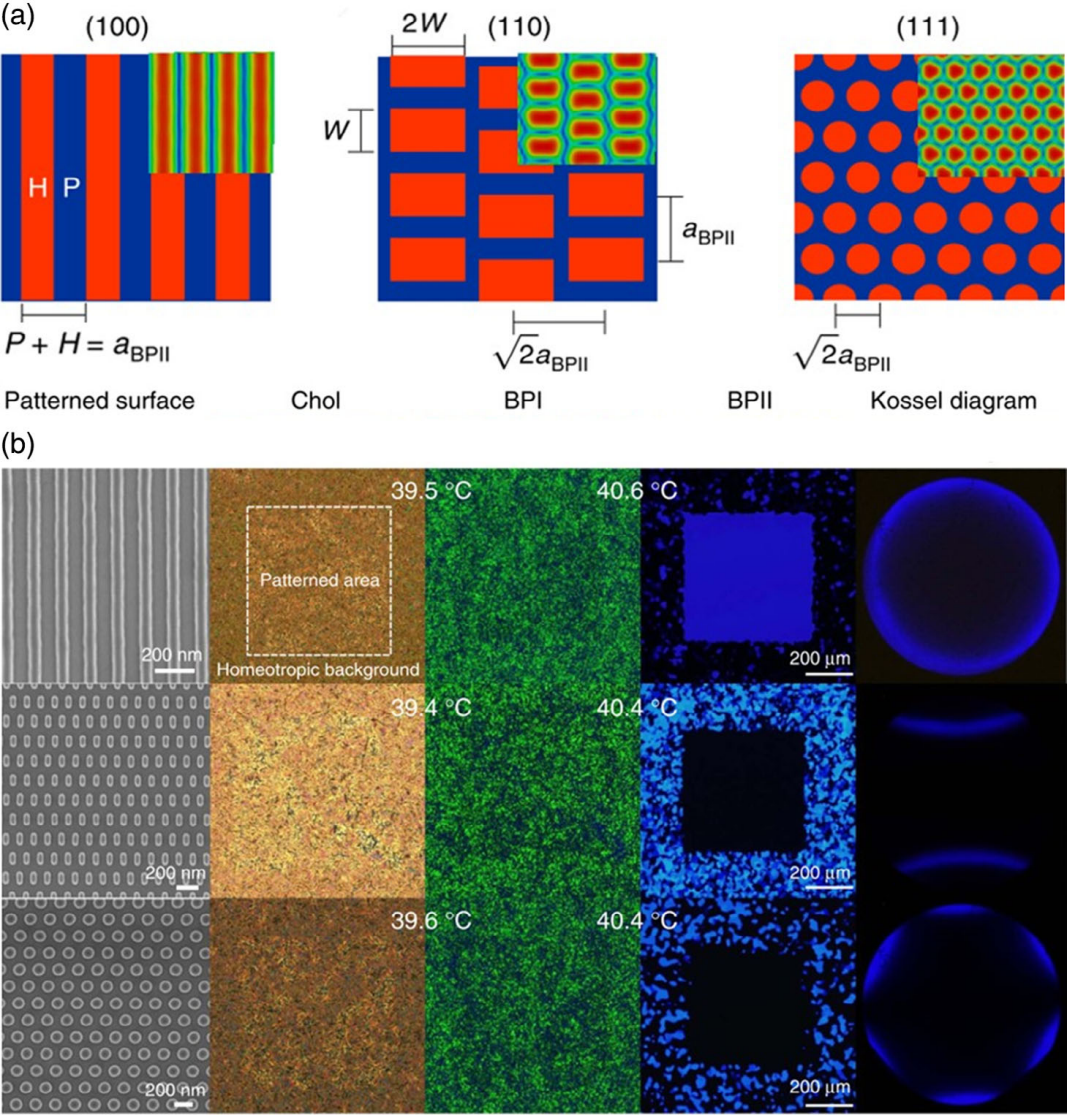
Macroscopic monocrystalline BPLCs formed by nanopatterned substrates. a) Patterned templates for monocrystalline BP II with different lattice orientations. b) SEM images of three different patterned surfaces, the corresponding POM images of the cholesteric phase, BP I, and BP II, and Kossel diagrams of patterned area. The resulting monocrystalline BPLCs are as large as the nanopatterned area. a,b) Reproduced under the terms of the CC‐BY 4.0 license.^[^ ^129^ ^]^ Copyright 2017, The Authors, published by Springer Nature.

Substrates with symmetric nanopatterns are conducive to the formation of monocrystalline BPLCs (BP I or BP II) having a definite crystallographic orientation.^[^ 129, 130, 131, 132, 136 ^]^ Martínez‐González et al. reported the directed self‐assembly of BP lattices into large‐scale monocrystalline BPLCs using well‐designed nanopatterned surfaces (**Figure** **4**).^[^ ^129^ ^]^ Nanopatterned substrates capable of directing and stabilizing the lattice orientation of BPLCs were judiciously designed by theoretical and simulation studies. Striped, rectangular, and circular nanopatterns were found to induce the formation of BP II (100), BP II (110), and BP II (111) monocrystals, respectively (Figure 4a). BP II (110) and BP II (111) monocrystals appeared black under a POM, as their selective reflections were located in the UV spectral range (Figure 4b). The different regions of the samples gave rise to the same Kossel pattern, indicating the formation of BP monocrystals. In contrast, the polycrystalline BP structures generated different Kossel patterns for different areas. The resulting monocrystalline BPLCs were as large as the nanopatterned area. Li et al. further studied stripe‐patterned substrates to produce large monocrystalline BPLCs.^[^ ^131^ ^]^ The pattern periodicity may affect the nucleation and growth of BP soft crystals as these 3D photonic crystals have submicronic lattice parameters. Therefore, the nanometric variation of pattern dimensions may affect the formation of BP monocrystals with a specific lattice orientation. The strategy of using nanopatterned substrates for fabricating macroscopic monocrystalline BPLCs is significantly faster than other approaches, and can be easily reproduced due to the regular pattern geometry. These properties are promising and essential for preparing soft 3D photonic crystals with ideal nanostructures.

**Figure 5:**
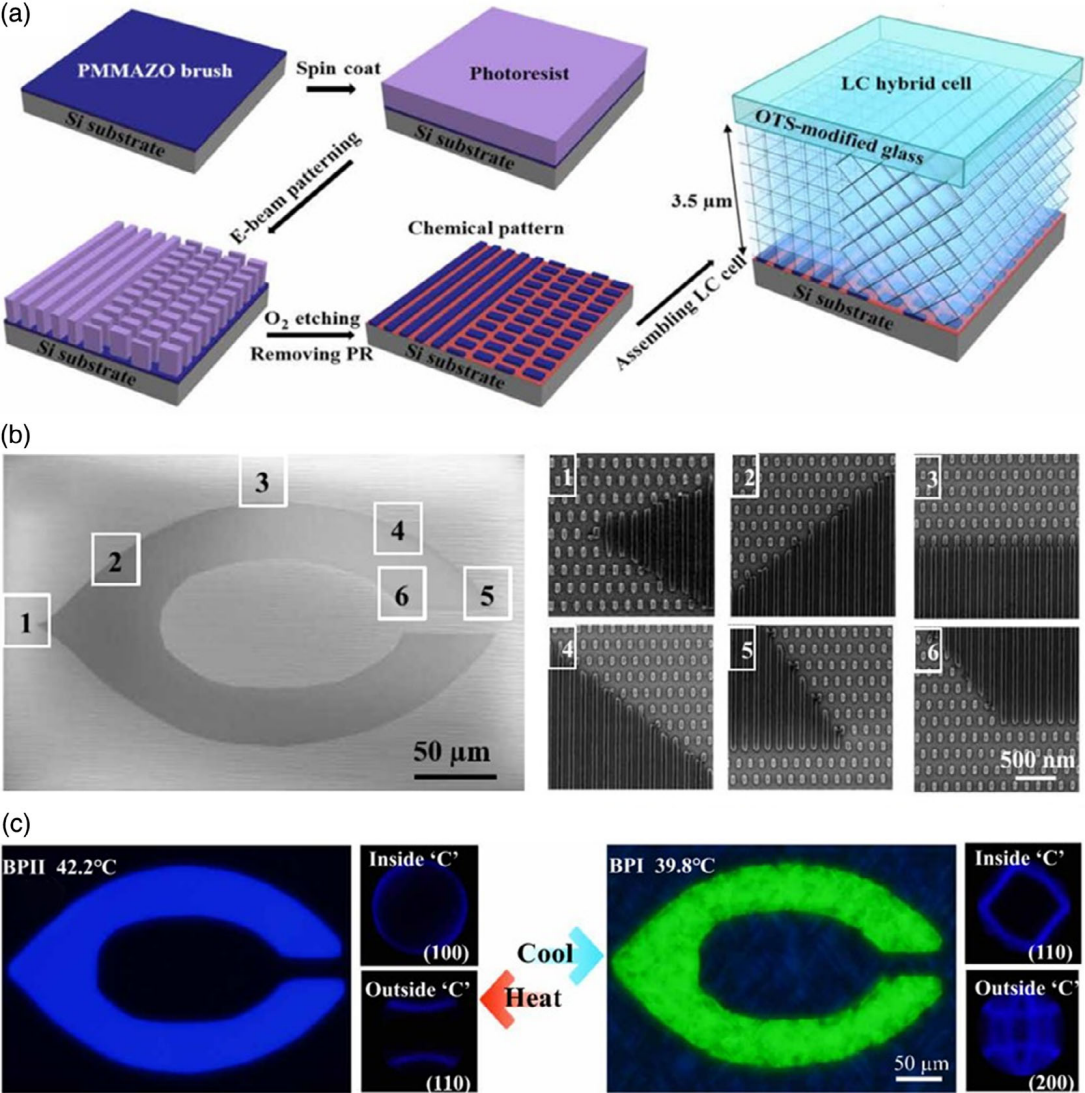
a) Fabrication processes of soft heteroepitaxy nanopatterns on a Si substrate. b) A “stripe pattern” surface designed as a “C” and a “rectangular pattern” designed in other regions. c) POM images of sculpted BP nanostructures with Kossel diagrams corresponding to BP II (100), or BP I (110), inside the “C” region and BP II (110), or BP I (200), outside. a–c) Reproduced with permission.^[^ ^132^ ^]^ Copyright 2019, American Association for the Advancement of Science.

To further explore the soft feature of BP‐based 3D photonic crystals in terms of grain boundaries, Li et al. introduced a “soft heteroepitaxy” strategy to manipulate the lattice orientation of BPLCs based on the sophisticated design of lithographically nanopatterned substrates (**Figure** **5**).^[^ ^132^ ^]^ It was found that the judiciously designed substrates with unique patterns at the nanoscale could result in the formation of BP‐based 3D soft crystal with predetermined lattice orientation in a desired manner. The BP‐based single crystals with a variety of in‐ and out‐of‐plane orientations could be easily achieved using appropriately patterned substrates, and the grain boundaries could be precisely controlled through using a substrate with two different pattern symmetries. It should be noted that precise control over the solid–solid grain boundary or liquid–liquid interfaces is a challenging task. Liquid–liquid interfaces are particularly attractive due to their capacity to induce self‐assembly, including nanoparticle self‐assembly,^[^ ^134^ ^]^ supramolecular self‐assembly,^[^ ^135^ ^]^ and ion transfer.^[^ ^136^ ^]^ Thanks to the liquid‐crystal nature of the BPs, it is possible to fully overcome challenges that are traditionally associated with manipulation of solid crystalline materials, and the limited grain morphologies accessible with liquid–liquid interfaces. The structured liquids with an unprecedented precision of grains, distortions, and arbitrary shape could not only open new opportunities for the design and fabrication of dynamically transformable soft 3D photonic crystals, but also deepen our understanding of fundamental science in a variety of complicated interfacial processes.

### 3.2 Lattice Orientation under Electric Field

**Figure 6:**
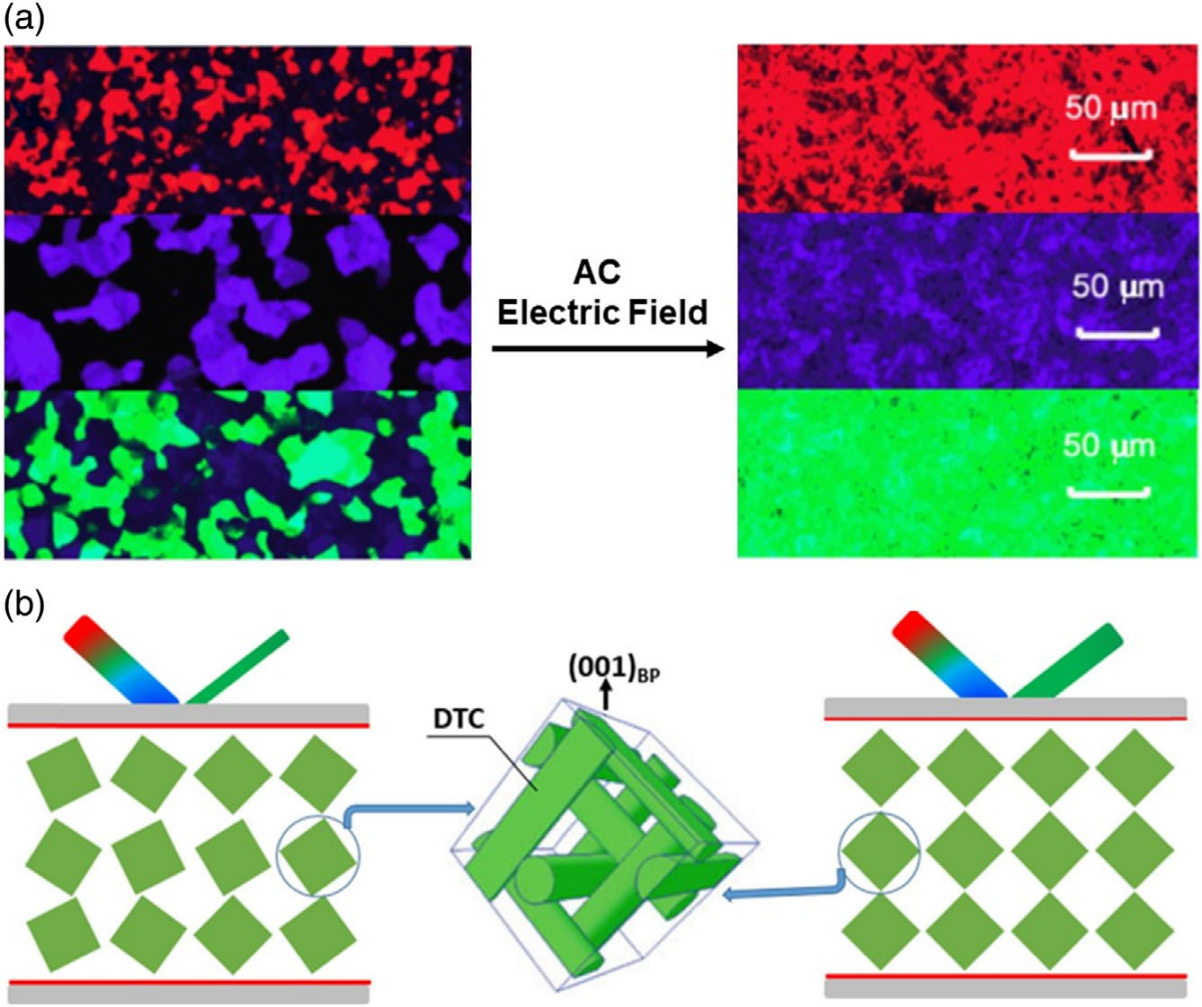
Monodomain BPLCs formed under an applied electric field. a) POM images of multidomain BPLCs and electric field‐induced monodomain BPLCs. Reproduced with permission.^[^ ^137^ ^]^ Copyright 2013, AIP Publishing. b) Schematic of the underlying mechanism of electric field‐induced monodomain formation in BPLCs. Reproduced with permission.^[^ ^141^ ^]^ Copyright 2019, American Chemical Society.

Monodomain BP textures can be induced by rotating the BP lattices under an electric field.^[^ 137, 138, 139, 140, 141, 142 ^]^ Chen and Wu prepared monodomain BPLCs by applying an alternating current electric field, as shown in **Figure** **6**a.^[^ ^137^ ^]^ POM analysis revealed multidomain platelet textures, indicating the random alignment of BP lattices before the application of an electric field. When an alternating current electric field of 2 V μm^−1^ was applied to the sample for about 1 s, the BP lattices oriented along the electric field, forming monodomain BPLCs with uniform textures. Compared with a multidomain structure, the monodomain BPLCs exhibited a sharp bandgap and considerably higher reflectivity, thereby exhibiting vivid colors. Moreover, the reflectivity gradually decreased with increasing operating voltage, and the corresponding grayscales were obtained. Du et al. prepared electric field‐induced monodomain BP nanostructures with a high reflectivity (Figure 6b).^[^ ^141^ ^]^ The BP lattices, which were randomly oriented before the electric field was applied, oriented uniformly after the application of an electric field. The self‐organized monodomain BP films have potential application in 3D tunable lasers, nonlinear optics, and high‐performance full‐color reflective displays. Yan et al. found that the nucleation and growth of BPLCs could be affected by the strength and frequency of the electric field.^[^ ^142^ ^]^ Crystal growth occurred through heterogeneous nucleation at a predetermined applied voltage, and the morphology of the BPLCs could be precisely controlled by modulating the applied voltage. After polymerization, uniform monodomain PSBPs were achieved and their stable temperature range was extended to 70 °C (from 0 to 70 °C) including the room temperature. This work provided a significant insight into BP crystal growth and lattice orientation under an applied electric field. Although monodomain BPLCs can be easily prepared by applying an electric field, the BP lattices are well oriented only in the crystallographic axis along the electric field and are randomly oriented in other directions. This remains a challenge in the preparation of monocrystalline BPLCs by applying an electric field.

**Figure 7:**
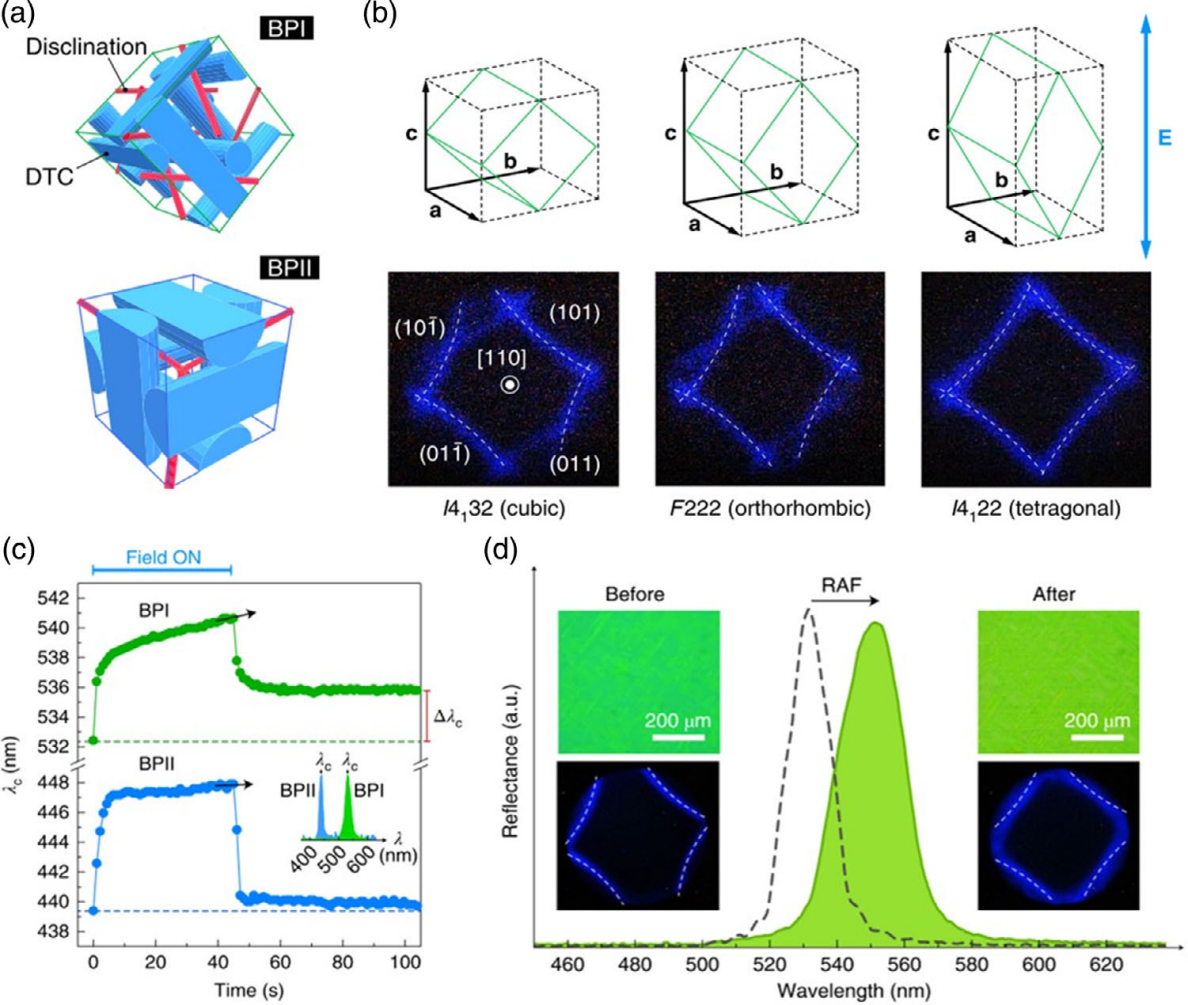
a) Schematic description of the unit cells of BP I and BP II. Within the BP lattice, the disclinations are detached from each other in BP I but interconnected in BP II. b) Schematics and Kossel diagrams of cubic, orthorhombic, and tetragonal lattices with the (110) direction parallel to the electric field. c) Time evolution of photonic bandgaps in BP I and BP II under a 4 V μm^−1^ alternating current electric field applied for 45 s. d) Reflection spectra, optical images, and Kossel diagrams of RAF‐treated BPLCs with different pitches. a–d) Reproduced with permission.^[^ ^143^ ^]^ Copyright 2020, Nature Publishing Group.

Electric field can induce the reconfiguration of BP lattices into new stable photonic crystal lattice structures. Recently, Guo et al. used repetitive electrical pulses to reconfigure BPLCs into stable tetragonal and orthorhombic lattices (**Figure** **7**).^[^ ^143^ ^]^ Within a BP lattice, the disclination lines are detached from each other in BP I but interconnected in BP II (Figure 7a). Because of the difference in defect arrangement within the unit cells of BP II and BP I, the electrostriction dynamics in BP II and BP I are strikingly different. In the case of BP II, the shifted peak returned to its original position when the applied electric field was removed after 45 s, indicating restoration to the initial lattice. In the case of BP I, the initial fast build‐up was followed by a stable and slight redshift (Δ*λ*_c_ ≈ 3.4 nm), which implies residual lattice distortion (Figure 7b,c). A repetitively applied field (RAF) technique was further proposed, which allowed the system to relax after each pulse, gradually deforming the BP I cubic lattice into various intermediate metastable states until stable noncubic BP crystals were formed (Figure 7d). This technique is suitable for engineering cubic crystals into noncubic lattices with tailored reflective wavelengths in the entire visible spectrum. These field‐free BPLCs exhibited fast electro‐optic responses and are promising materials for application in electro‐optics, information display, microlasers, nonlinear optics, and biosensing technologies.

### 3.3 Lattice Orientation by Controlling Self‐Assembly Processes

**Figure 8:**
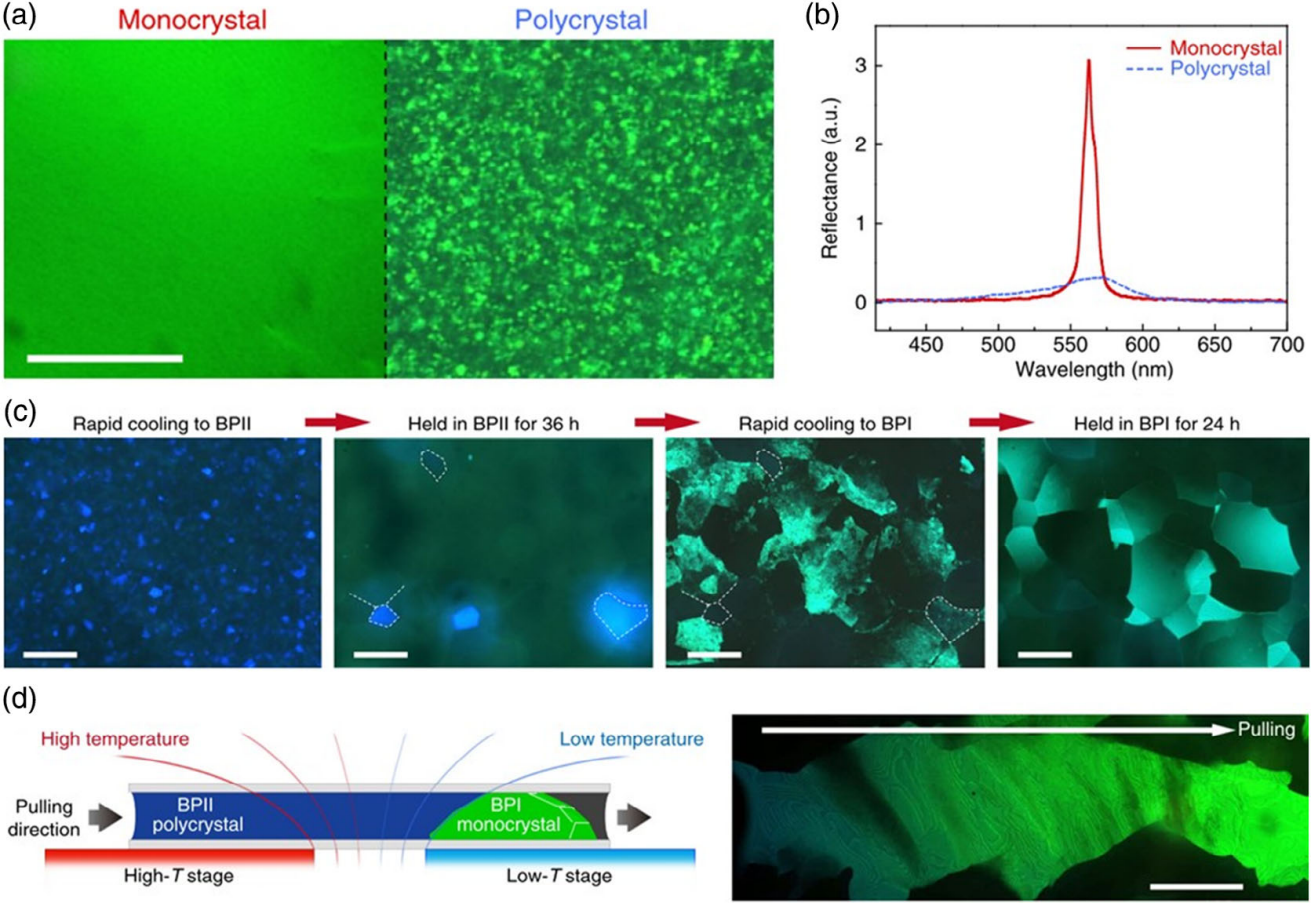
Formation of large monocrystalline BPLCs by controlling self‐assembly processes. a) Microscope images of BPI monocrystal and polycrystal (scale bar, 100 μm). b) Reflection spectra of BP I monocrystal and polycrystal. c) Microscopic images of the sample upon cooling from isotropic phase to BP II, holding in BP II for 36 h, cooling to BP I, and holding in BP I for 24 h (scale bars, 300 μm). d) Formation of large monocrystalline BP I by gradient‐temperature scanning. a–d) Reproduced under the terms of the CC‐BY 4.0 license.^[^ ^144^ ^]^ Copyright 2017, The Authors, published by Springer Nature.

Monocrystalline BPLCs can also be prepared by controlling the self‐assembly process. Chen et al. prepared macroscopic monocrystalline BPLCs with a uniform POM texture, higher reflectance, and narrower photonic bandgaps compared with those of polycrystalline BPLCs (**Figure** **8**a,b).^[^ ^144^ ^]^ They found that the crystalline domain size of BP I platelets barely changed when held at the BP I state temperature, whereas the domain sizes of BP II gradually increased when held at the BP II state temperature (Figure 8c). The difference in the growth of large crystals in BP I and BP II can be attributed to the presence of independent disclination lines within the lattice of BP I, and interconnected disclination lines that to merge into a single defect within BP II. Therefore, large BP II monocrystals with a lateral size of ≈1 mm could be prepared by holding at the BP II state temperature for 1 week. A gradient‐temperature scanning technique was further invented (Figure 8d). One of the heating stages was set as the BP II temperature (high‐T stage), whereas the other was set as the BP I temperature (low‐T stage). A temperature gradient was generated by placing the cell across the two stages, and a micrometer step motor was used to move the sample from the high‐T stage to the low‐T stage, ensuring a controllable BP II‐to‐BP I phase transition of the sample. Finally, a remarkably large monocrystalline BP I was prepared from a pregrown BP II single crystal. These macroscopic single crystals exhibited long‐range lattice orientation in all dimensions and remarkably narrow photonic bandgaps with high reflectivity.

**Figure 9:**
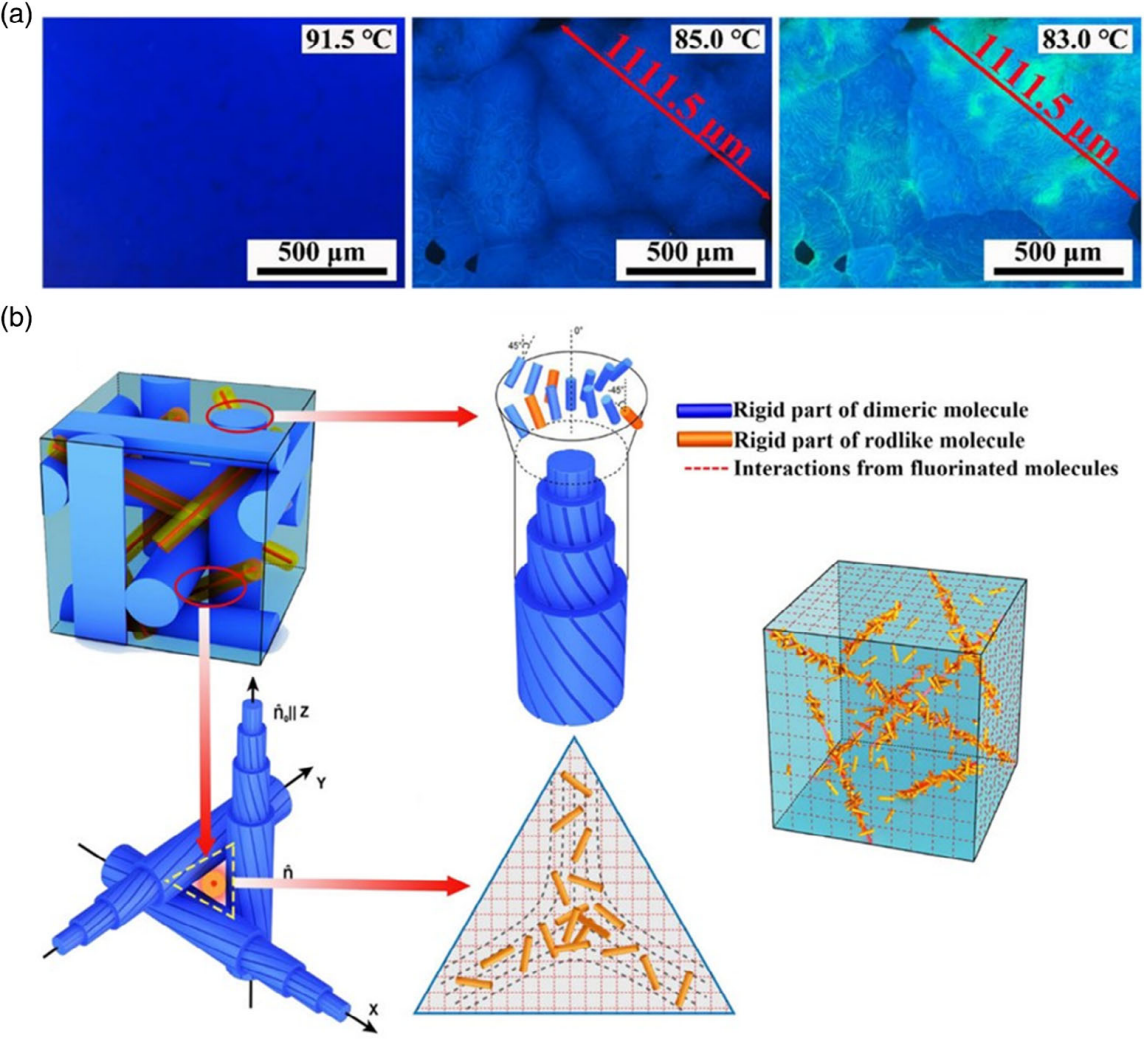
Formation of large monocrystalline BPLCs by molecular synergistic self‐assembly. a) Large BP single crystals obtained via thermal treatment. b) Schematic illustration of stabilizing BP by molecular synergistic self‐assembly. The defect region filled by rodlike molecules. a,b) Reproduced under the terms of the CC‐BY 4.0 license.^[^ ^145^ ^]^ Copyright 2021, The Authors, published by Springer Nature.

Recently, Hu et al. found that large monocrystalline BPLCs with broad temperature range could be achieved by a molecular synergistic self‐assembly of biaxial and uniaxial mesogens.^[^ ^145^ ^]^ A series of tailored uniaxial rodlike mesogens was introduced into a BP system comprising biaxial dimeric mesogens, BPLCs with stable 3D nanostructures were observed continuously over a ultrawide temperature range of 132.0 °C, from 92.8 to −39.6 °C. Large monocrystalline BPLCs with the diameter of the maximum platelet exceeding 1100 μm (**Figure** **9**a) could be obtained from such unique system through a thermal treatment process upon cooling the sample in a 20.0 μm‐thick cell with no initial surface treatment. The results indicate that the judiciously designed molecular system could exhibit a low interfacial energy with the liquid crystal (LC) molecules in the DTCs, which could efficiently fill the defects in the cubic lattice and reduce the total free energy, thus enhancing the stability of the BPLCs (Figure 9b). This molecular synergistic self‐assembly of a multicomponent system may also be an extremely attractive approach to developing other multifunctional soft nanomaterials.

## 4 Stimuli‐Driven BP Photonic Nanostructures

3D photonic nanostructures with tunable performances have received extensive attention due to their light manipulation capability in all the three dimensions. As soft 3D photonic crystals, BPLCs offer excellent reflection tunability over a broad spectral range in response to different stimuli such as temperature, electric field, light, magnetic field, and chemical solvents. Thus, the 3D nanostructures produce tunable reflective colors. The stimuli‐driven liquid‐crystalline BP nanostructures and the reversible tuning of their selective reflections is a fascinating research area due to their potential application in advanced technologies such as sensors, displays, tunable mirrorless lasers, switchable color filters, and micro‐/nanophotonics.

### 4.1 Thermoresponsive BPLCs

The reflective wavelengths of BP I are usually temperature independent because the BP platelets are pinned by boundary anchoring forces, which restricts bandgap variation. The photonic bandgaps of BP II slightly blue shift with a decrease in temperature.^[^ ^146^ ^]^ The BP II lattice unit is considered to deform upon cooling, and the shift in the reflective wavelength is usually less than 50 nm. In addition, the photonic bandgaps exhibit a discontinuous shift during BP II‐to‐BP I phase transition due to the changes in crystalline symmetry and orientation. Based on the thermo‐optical properties of BPLCs, Petriashvili and Chanishvili fabricated a thermal imaging device that exploits the attributes of phase transitions between BP I and BP II to show brilliant colors due to the different reflective wavelengths.^[^ ^147^ ^]^ At temperatures higher than the BP II‐to‐BP I phase transition temperature, the color of the thermal device changes from red to green. The thermal imaging device offers several advantages over traditional thermal imaging systems such as a high spatial resolution, low cost, low weight, and real‐time operation capability.

**Figure 10:**
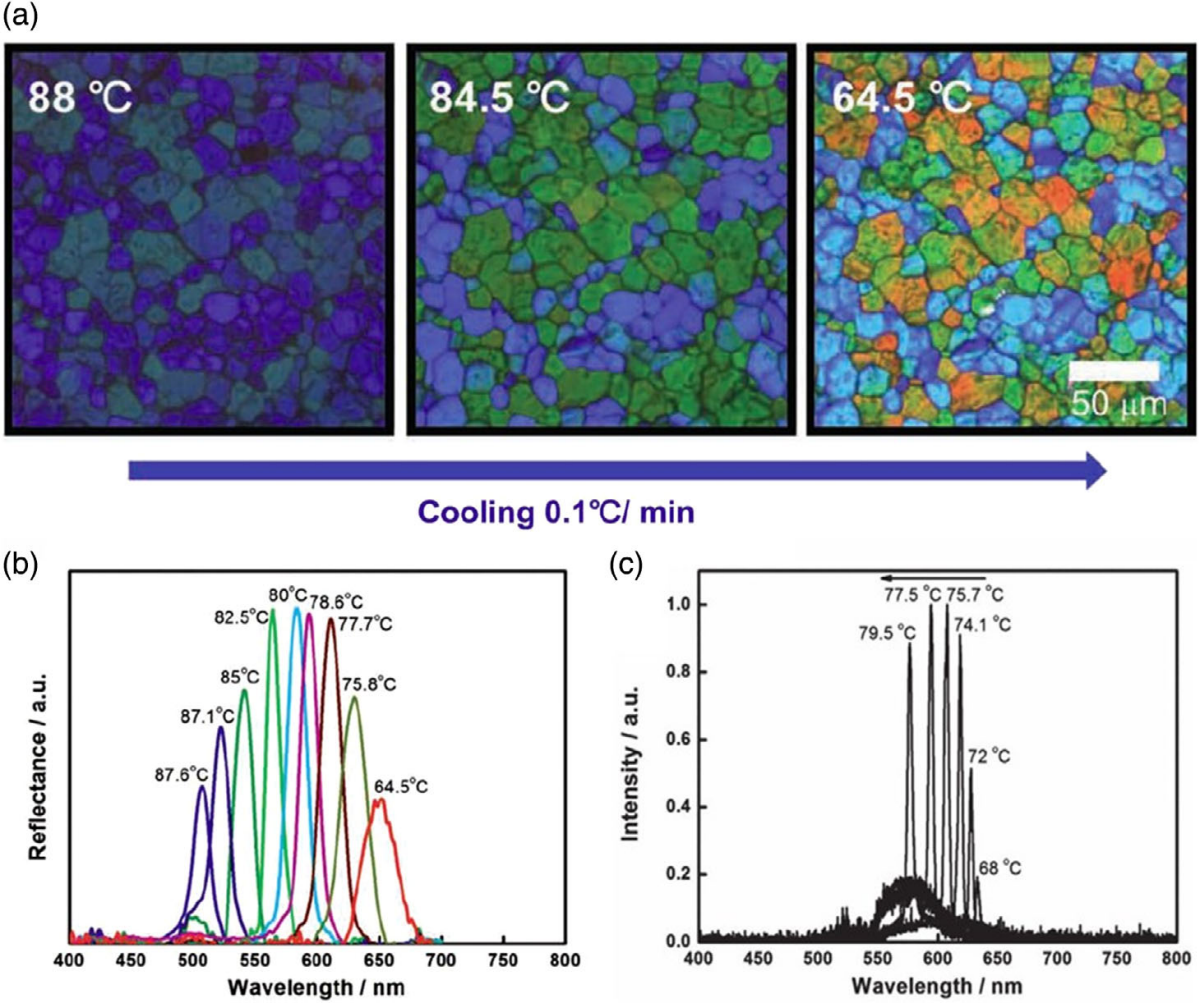
Temperature‐driven BPLCs with wide tunable photonic bandgaps. a) Typical POM images of BPLCs upon cooling from 88 to 64.5 °C b) Reflection spectra at different temperatures. c) Tunable laser emissions during cooling.^[^ ^148^ ^]^ a–c) Reproduced with permission. Copyright 2013, Wiley‐VCH.

Hur et al. designed a temperature‐responsive bent‐core mesogenic molecule that was incorporated into BPLCs to dynamically control their photonic bandgaps with temperature changes to achieve tunable laser emissions (**Figure** **10**).^[^ ^148^ ^]^ The BP was composed of temperature‐sensitive bent‐core mesogens, chiral dopants, and rod‐like liquid crystals. The reflection colors of the BP textures gradually changed from blue to red upon cooling as the reflection color was associated with the BP I (200) reflection of the cubic lattice vectors. A reversible and tunable selective reflection with a wide wavelength range of 150 nm was achieved when the temperature was decreased from 88 to 64.5 °C (Figure 10a,b) due to the unique temperature‐responsive property of the bent‐core mesogens. In addition, temperature‐driven reversible tuning of laser emissions was achieved over a wide spectral range (Figure 10c). To prepare BPLCs with temperature‐driven reversibly tunable reflections over a wide wavelength range, the BP lattices should be thermally deformable and the BPLCs should have a wide temperature range. These two features can be simultaneously achieved by incorporating temperature‐responsive bent‐core mesogens into a BP host.

### 4.2 Electroactive BPLCs

The most complicated but remarkably attractive way of controlling the photonic bandgaps of BPLCs is the application of electric fields. In general, BPLCs undergo three distinct transformations under an applied electric field: local director reorientation, lattice distortion, and field‐induced phase transition. In local director reorientation, also called the Kerr effect, the liquid‐crystal molecules tend to align with the electric field. The Kerr effect is the basic principle of next‐generation ultrafast liquid‐crystal displays with submillisecond switching speeds. Lattice distortion occurs when a strong electric field is applied to BPLCs, which often leads to the shifting of the photonic bandgap. The response speed in this case is much slower than that to local director reorientation, which can be attributed to the rearrangement and movement of disclinations within the 3D nanostructures. Compared with lattice distortion, field‐induced phase transition requires a much higher electric field strength and usually results in an irreversible and discontinuous shift in selective reflections.^[^ 149, 150, 151, 152, 153 ^]^

**Figure 11:**
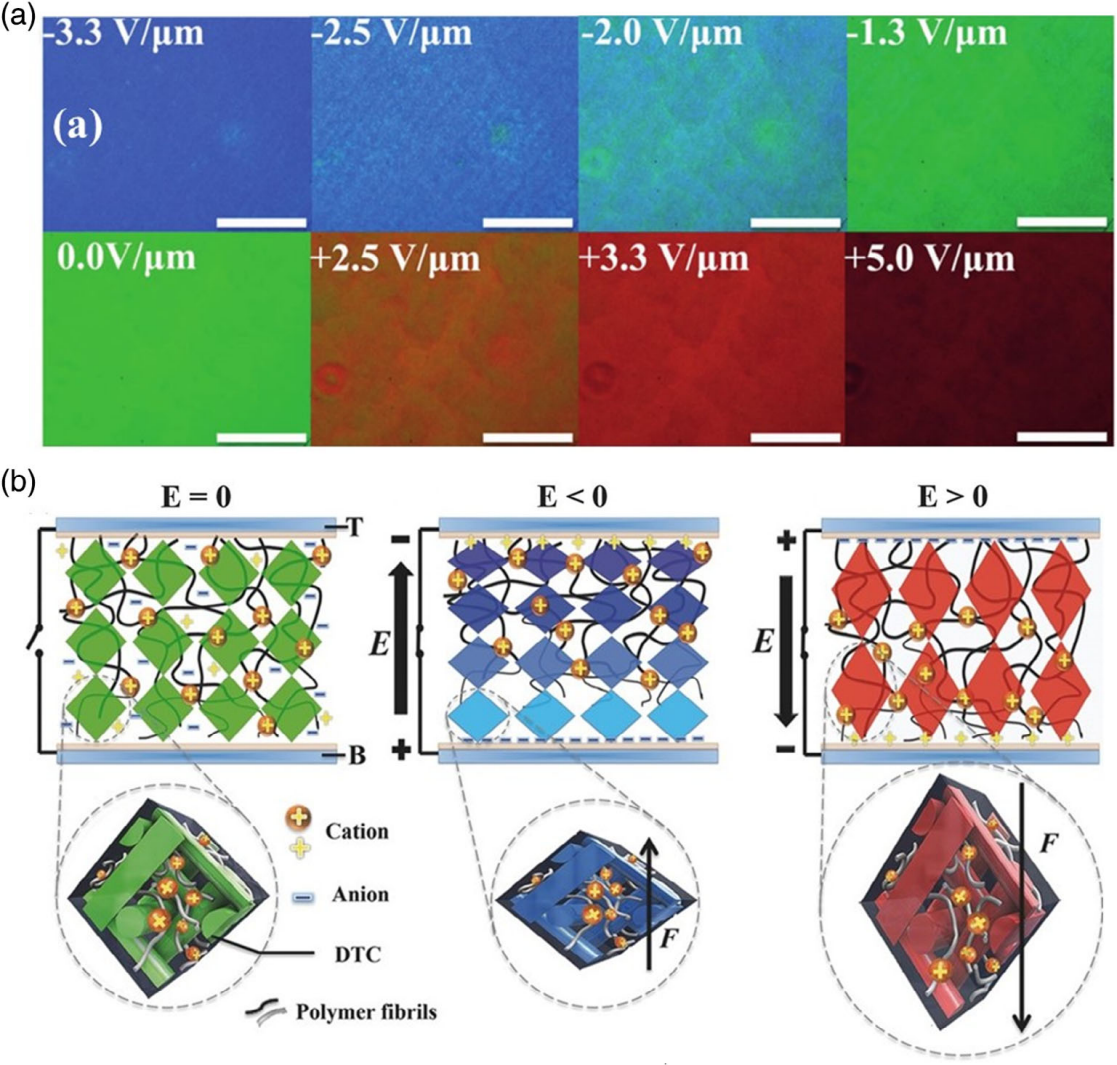
Electric field‐driven PSBPs with reversible tunable reflection colors. a) POM textures of the BP sample under various applied electric fields. b) Schematic of cubic nanostructures of the PSBP sample in the original state, blue‐shift state, and red‐shift state. a,b) Reproduced with permission.^[^ ^155^ ^]^ Copyright 2017, Wiley‐VCH.

Chen et al. reported that the electrically driven effect can arbitrarily manipulate the photonic bandgap of PSBPs through shifting or expansion under a direct current field.^[^ ^154^ ^]^ For a 12 μm‐thick liquid‐crystal cell, the selective reflections could be reversibly shifted within a wide spectral range exceeding 200 nm. The conduction of the positively charged ions trapped in the polymer network occurred under direct current fields, resulting in BP lattice transformation. Because of the confinement of liquid‐crystal molecules by the polymer network, only the lattice spacing along the electric field axis could be tuned, while those in the other dimensions remained unchanged. In the case of a 27 μm‐thick liquid‐crystal cell, the application of a direct current field to the film expanded the wavelength range of selective reflections instead of shifting them. The application of a 0.89 V μm^−1^ electric field extended the bandwidth of the reflection band from 34 to 310 nm, involving almost the entire visible range. Similarly, Wang et al. investigated electrically manipulated tunable photonic bandgaps of PSBPs in the visible spectrum by controlling the strength and polarity of the direct current field (**Figure** **11**a).^[^ ^155^ ^]^ The selective reflections reversibly blue shifted under a negative bias but red shifted under a positive bias due to the lattice distortion of the cubic nanostructure induced by the mobility of polymer networks under the direct current field (Figure 11b). Moreover, the self‐assembled PSBP samples were used in electrically tunable lasers by doping with fluorescent dyes. This demonstrates the enormous application potential of the PSBPs in intelligent electro‐optical devices.

**Figure 12:**
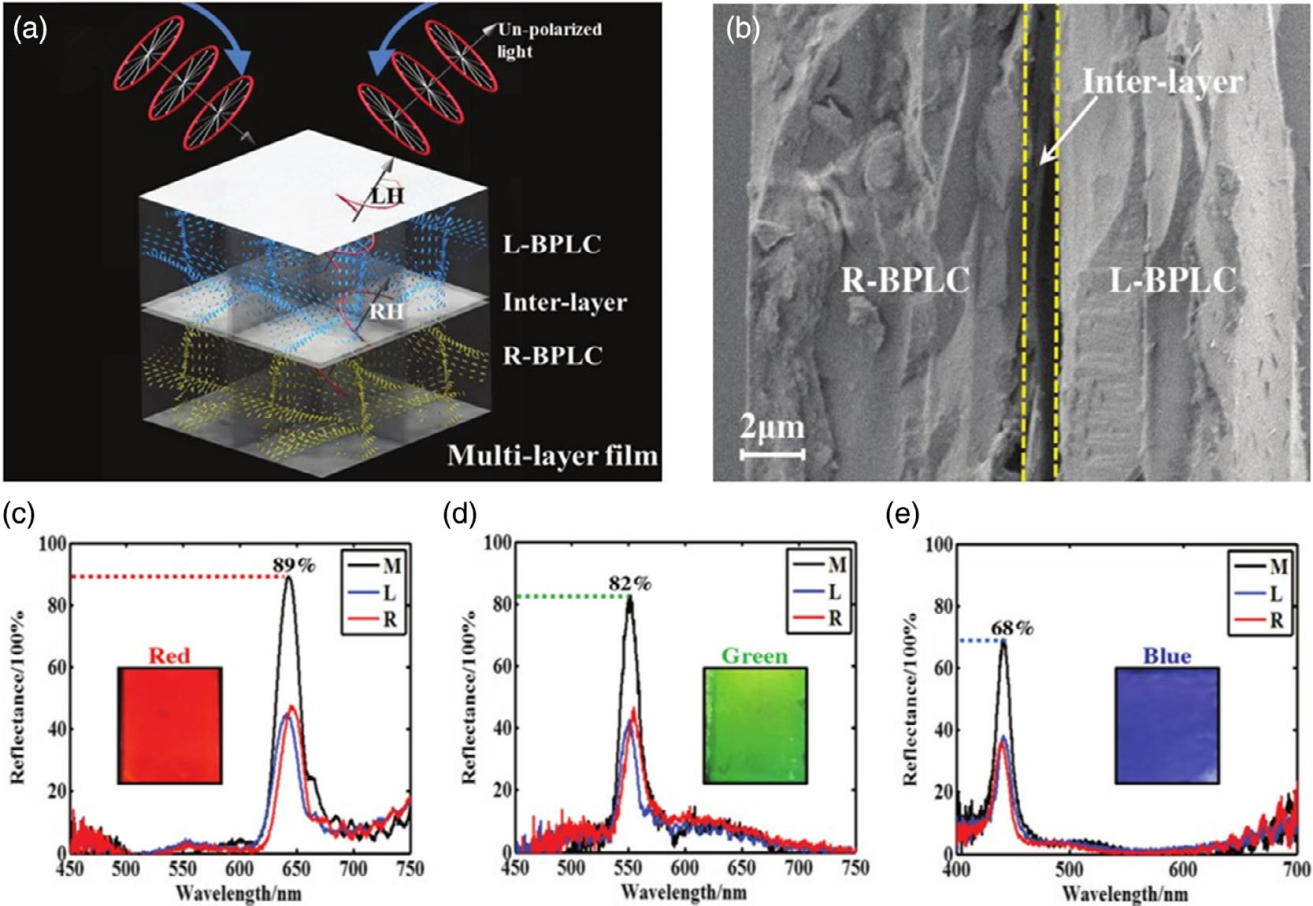
Electrically switchable, hyper‐reflective BP liquid‐crystalline films. a) Hyper‐reflective film comprising three layers: left‐handed BP layer, interlayer, and right‐handed BP layer. b) SEM image of cross section of the multilayered BP film. Reflection spectra of multilayer BP films with c) red, d) green, and e) blue reflective colors. The insets show the photographs of the BP films with bright colors. a–e) Reproduced with permission.^[^ ^156^ ^]^ Copyright 2017, Wiley‐VCH.

BP is a helical superstructure with either left‐handed or right‐handed molecular organization. Therefore, single‐layer BP films have a reflectivity of up to 50%, which limits their application in a wide range of fields. Xu et al. reported electrically switchable, hyper‐reflective, and fast‐responsive displays based on multilayer BP films containing two single‐layer BP templates with opposite handedness and an interlayer between them (**Figure** **12**a,b).^[^ ^156^ ^]^ Red, green, and blue films with hyper‐reflectivities of 89, 82, and 68% were obtained by refilling achiral nematic liquid crystals into the multilayer BP templates (Figure 12c). In addition, reflectance switching of the film was realized by the local director reorientation of the liquid‐crystal molecules in a 1 V μm^−1^ electric field. The highest reflectance obtained for the red, green, and blue films were 94, 86, and 72%, respectively. The electric field‐driven hyper‐reflective BP films are a promising platform for next‐generation devices, including switchable optoelectronic devices and sequential colorful reflective displays.

### 4.3 Light‐Driven BPLCs

**Figure 13:**
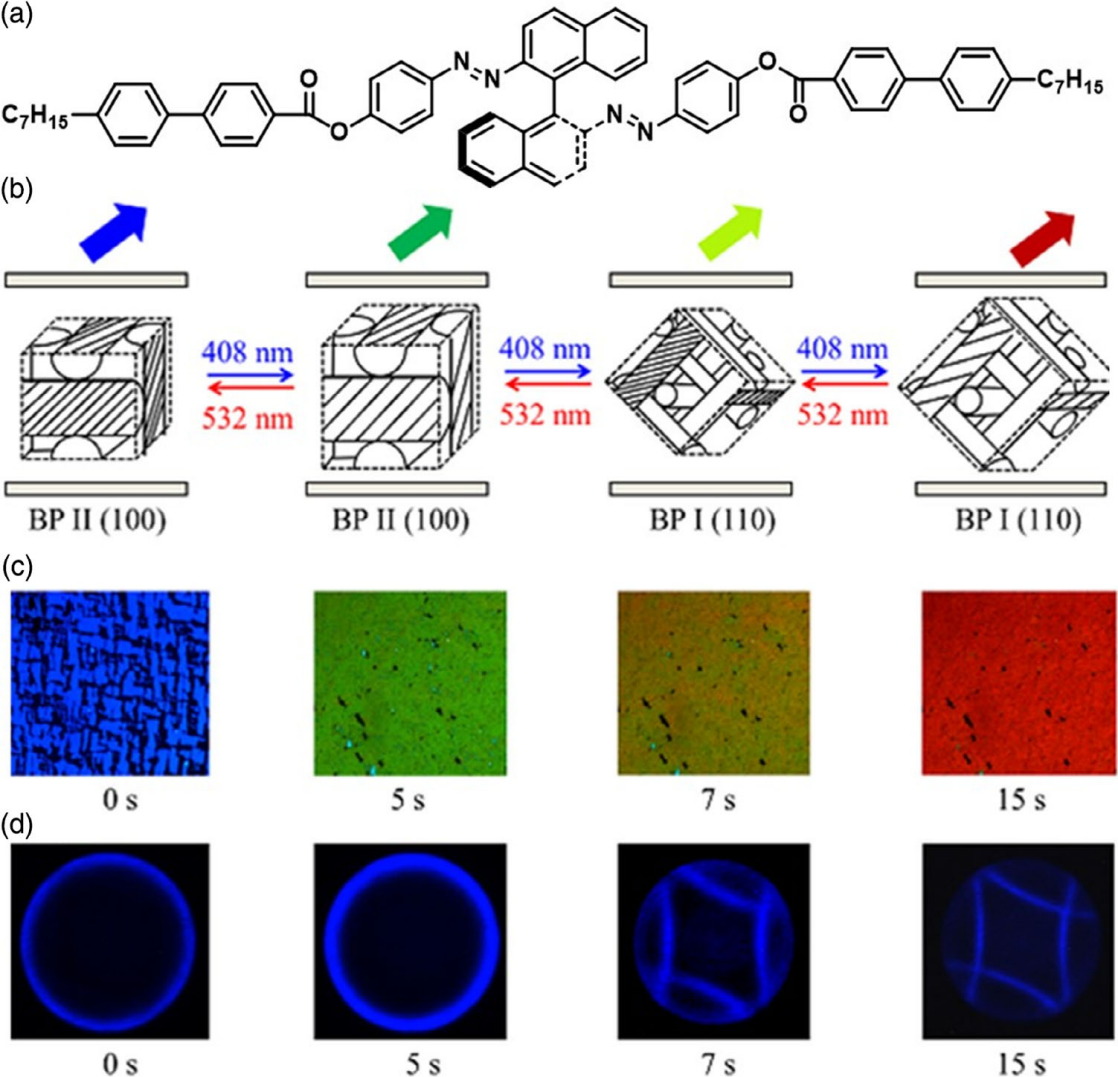
Light‐driven photonic bandgap shifting of BPLCs. a) Chemical structure of light‐driven chiral azobenzene molecular switch. b) Schematic of cubic nanostructures reversibly switched by light. c) POM images of BPLCs irradiated with 408 nm light. d) Kossel diagrams. a‐d) Reproduced with permission.^[^ ^160^ ^]^ Copyright 2013, Wiley‐VCH.

Much effort has been devoted to the phototuning of reflection colors and 3D architectures of BPLCs as light can be locally, remotely, temporally, and spatially controlled. So far, the light‐driven bandgap shifting of BPLCs has been mainly realized by doping BP liquid‐crystalline composites with chiral or achiral molecular switches such as azobenzene. The phase behavior of the resulting BPLCs can be efficiently manipulated by *trans*–*cis* photoisomerization of the azobenzene dopant.^[^ 157, 158, 159 ^]^ Lin et al. prepared a chiral azobenzene molecular switch and incorporated it into a BP host to realize the light‐driven shifting of photonic bandgap (**Figure** **13**).^[^ ^160^ ^]^ The resultant BP materials initially exhibited a blue reflection color and simple cubic BP II nanostructures. Upon 408 nm light irradiation, the reflection color of the BP sample turned red within 15 s due to the photoisomerization of the chiral azobenzene molecular switch. Kossel diagrams revealed that the original blue reflection originated from the BP II (100) lattices, whereas the yellow and red reflections originated from the BP I (110) lattices. The reverse process could be realized with 532 nm light irradiation. These light‐driven soft BP cubic superstructures are a promising prototype for the development of light‐driven 3D photonic materials for application in next‐generation optical devices.

**Figure 14:**
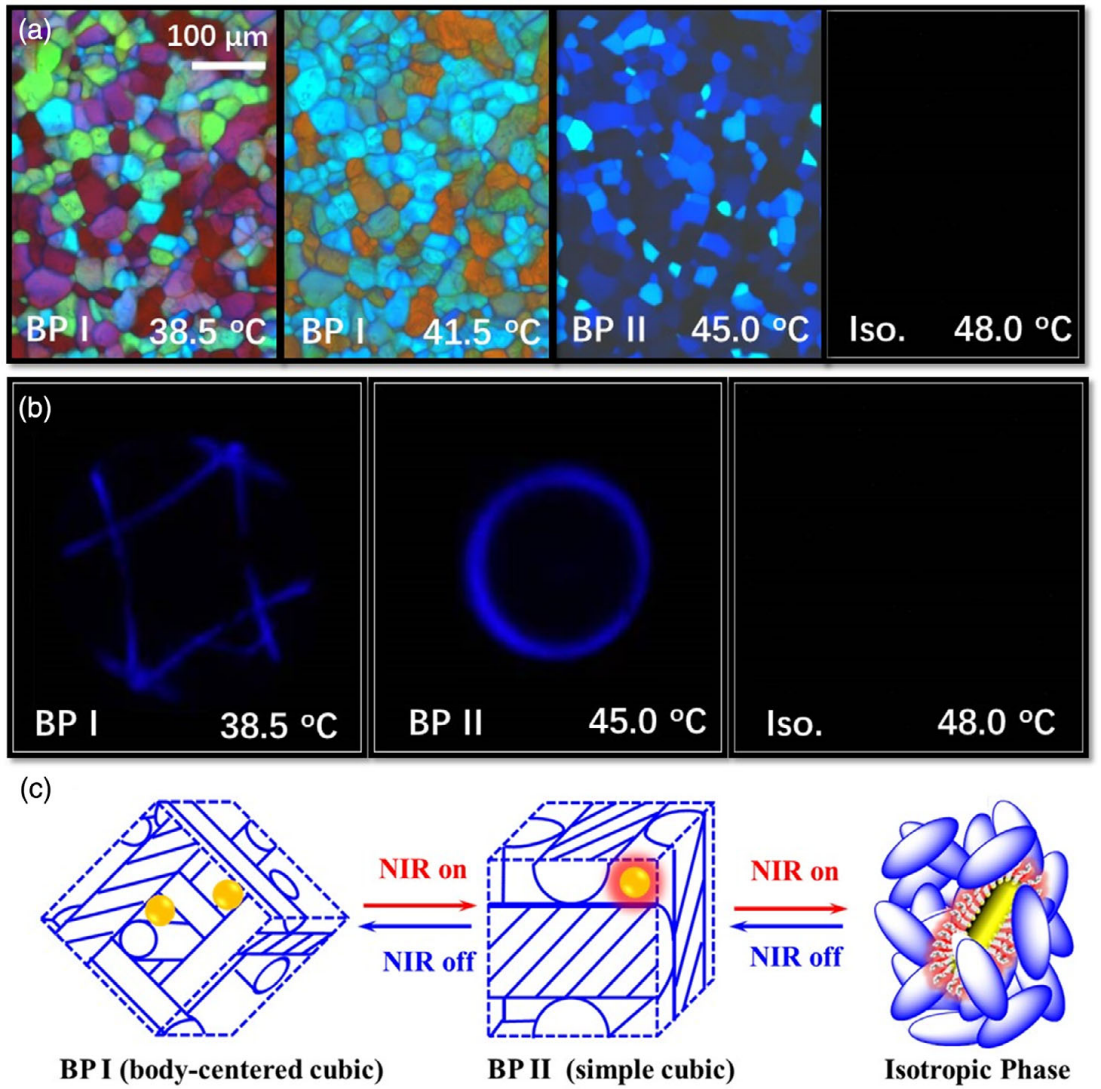
NIR light‐driven soft 3D BP photonic nanostructures. a) POM images of BP I, BP II, and isotropic phase in different temperatures. b) Corresponding Kossel diagrams. c) Schematic of phase transition induced by photothermal effect of the gold nanorods under NIR light irradiation. a‐c) Reproduced with permission.^[^ ^161^ ^]^ Copyright 2015, Royal Society of Chemistry.

However, the irradiation of liquid‐crystalline materials with high‐energy UV light may sometimes cause photodamage, photochemical contamination, or component degradation as well as poor penetration through the samples. Thus, near‐infrared (NIR) light has been explored due to its excellent penetration and invisibility for remote control. Wang et al. prepared a NIR light‐driven soft BP photonic nanostructure by doping a BP composite with novel surface‐functionalized gold nanorods (**Figure** **14**).^[^ ^161^ ^]^ Upon 808 nm NIR laser irradiation, the resultant BP nanostructures underwent phase transition from the simple cubic symmetry of BP II to the body‐centered cubic symmetry of BP I due to the significant photothermal effect of the embedded gold nanorods (Figure 14c). The reverse transformation occurred when NIR light irradiation was stopped. Moreover, the photonic bandgaps can be reversibly manipulated over a wide spectral range in such NIR‐light‐directed soft 3D photonic crystals. The research could provide new impetus to the manipulation of soft 3D photonic nanostructures by NIR light for photonic applications particularly in the field of life sciences.^[^ 162, 163 ^]^

### 4.4 Others

Magnetic field‐responsive materials have aroused enormous interest for technical applications due to their attractive merits such as outstanding performance, fast response, and energy saving. The most frequently used magnetic components are Fe_3_O_4_ nanoparticles, which can be easily synthesized and directly manipulated by applying a magnetic field. He et al. prepared a magnetic field‐driven BP material by doping a BP composite with surface‐functionalized Fe_3_O_4_ nanoparticles.^[^ ^164^ ^]^ The magnetic field‐driven BP materials exhibited a relatively higher contrast ratio in the BP state than in the isotropic‐phase state or cholesteric‐phase state, which could be attributed to the intrinsic features of the BP such as visible‐light reflection, moderate viscosity, and independent surface orientation. The patterns could be written with a magnetic pen and erased by applying a magnetic field to the opposite surface of the devices. The facile, efficient, and low‐cost strategy for preparing magnetic field‐responsive BPLCs is a promising method for the development of energy‐saving magnetic field‐driven flexible displays.

Materials that can monitor the density of environmental volatile organic compounds (VOCs) hold great promise for commercial, industrial, and personal applications. Yang et al. determined the response of PSBPs to VOCs (using toluene as a model compound) and compared it with the optical responses of BPLCs without polymer stabilization and polymerized cholesteric and nematic phases.^[^ ^165^ ^]^ They found that compared with polymerized cholesteric and nematic phases, PSBPs generated a greater response (change in reflection intensity) to toluene vapor with the limit of detection (140 ± 10 ppm at 25 °C) close to the permissible human exposure limit. In the PSBPs, the introduction of a guest (i.e., polymer) into the defects of the BP systems not only stabilized the BPLCs, but also enabled interaction with the targeted molecular species to realize high levels of selectivity. Overall, the thermal and mechanical stability of PSBPs enabled the passive detection of VOCs, which makes PSBPs a promising material for application in wearable and passive sensors for VOC detection.

Stimuli‐responsive liquid crystals confined within micrometer‐scale spaces have been developed as a platform for emerging technologies ranging from electro‐optical devices to biological sensors.^[^ 166, 167 ^]^ BPs are an especially appealing liquid‐crystalline state that can be confined within microdroplets as the selective reflections of BPLCs can be readily modulated to achieve high responsiveness to tune their optical properties. Bukusoglu et al. prepared aqueous dispersions of BP droplets and studied the effect of confinement on their spatial configuration and optical properties.^[^ ^168^ ^]^ They found that the BP droplets easily formed monodomain structures. The BP lattice spacings confined within the spherical monodomains could be strained, resulting in a change in the reflection colors of the droplets. The addition of amphiphiles to aqueous dispersions of BP droplets affected the BP lattice size, which changed the wavelength of light diffracted by these thermotropic microdroplets. The results suggest that BP droplets can be an appealing platform for responsive soft photonic materials for application in novel stimuli‐driven photonic technologies or low‐cost diagnostic devices.

## 5 Free‐Standing BP Polymer Films

BPLCs offer extraordinary advantages over their chiral liquid‐crystalline counterparts, such as narrow photonic bandgaps, unique 3D liquid‐crystalline nanostructures, and obviating the need for alignment. Traditional BP materials are kept in liquid‐crystal cells, which limit their optical performance. Although PSBPs have gained significant attention and are frequently used in electro‐optic devices with a wide temperature range, the content of polymer network is too low (less than 10 wt% monomers) for the materials to be free‐standing.^[^ ^96^ ^]^ Free‐standing liquid‐crystalline polymers can be fabricated by replacing liquid‐crystal molecules with reactive mesogens. The reactive mesogens, together with a photoinitiator, can be mixed and self‐organized with a liquid‐crystalline mixture; upon UV light exposure, a liquid‐crystalline polymer network is formed. Free‐standing BP photonic polymer films not only overcome the limitations of liquid‐crystal cells, but also exhibit unprecedented and superior optical performance.

**Figure 15:**
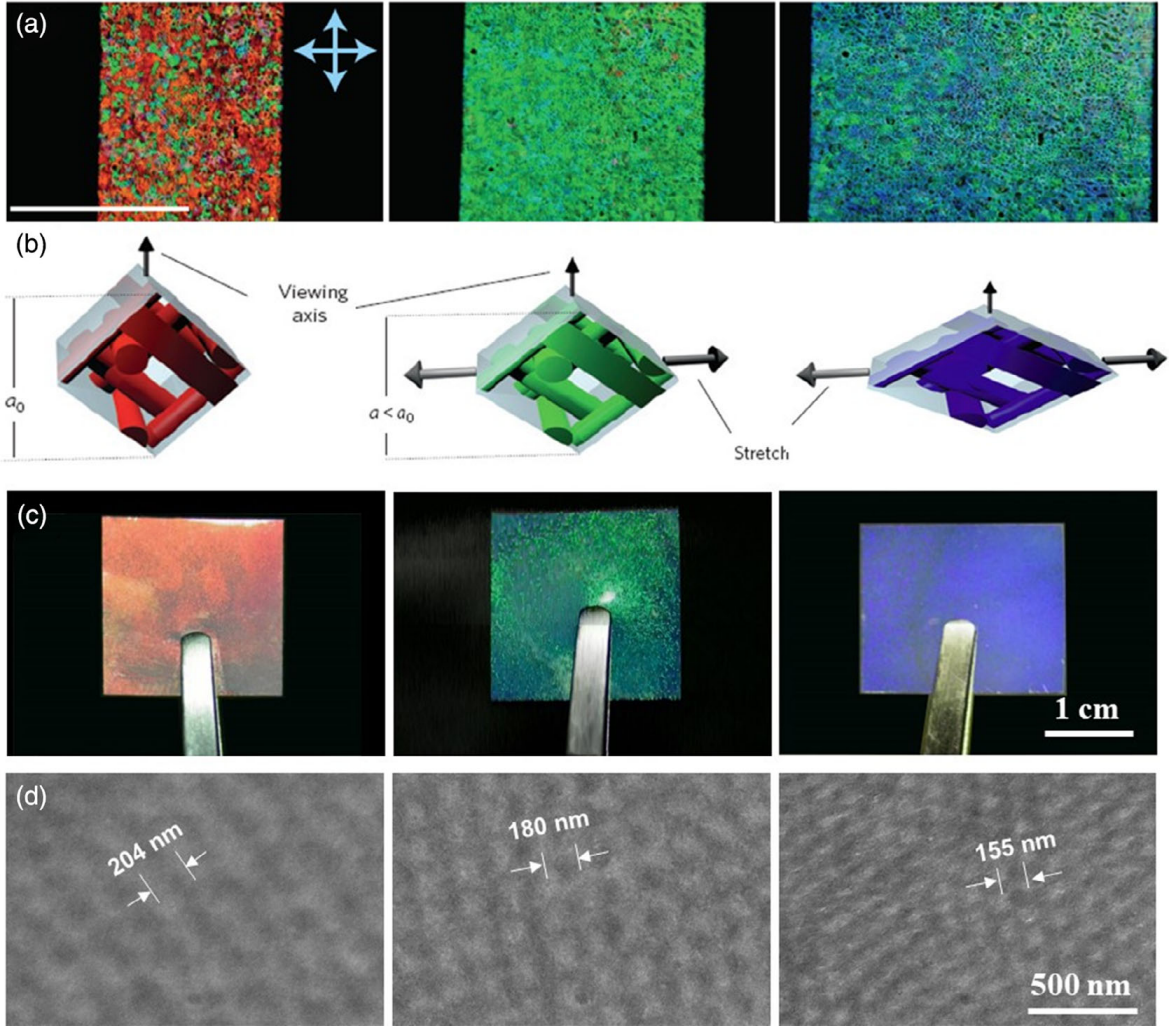
a) POM images of a BP film upon stretching. b) Schematic of the possible transformation of cubic BP I lattices. a,b) Reproduced with permission.^[^ ^113^ ^]^ Copyright 2014, Springer Nature. c) Photographs and d) microstructures of free‐standing BP films. c,d) Reproduced with permission.^[^ ^170^ ^]^ Copyright 2019, American Chemical Society.

Castles et al. reported a groundbreaking achievement of transferring soft BP materials into a free‐standing BP liquid‐crystalline film.^[^ ^112^ ^]^ The BPLCs were prepared by the photopolymerization of liquid‐crystal dimer materials with 3–5 wt% chiral dopant, 25–50 wt% reactive mesogen, and photoinitiator into a BP I state. Subsequently, the nonreactive mesogen was removed to obtain a free‐standing polymer scaffold as a 3D nanostructure template, whose chiral 3D BP structure was maintained by refilling with achiral liquid crystals. Because of the presence of 25–50 wt% polymer network in the system, the free‐standing BP liquid‐crystalline films exhibited unprecedented stability at temperatures over 250 °C. Castles et al. further constructed stretchable BP liquid‐crystalline gels, whose photonic bandgaps could be manipulated by applying an external strain (**Figure** **15**a,b).^[^ ^113^ ^]^ The gels exhibited a stress‐responsive behavior under an applied lateral stretch due to a decrease in film thickness, which decreased the valid BP lattice scale along the viewing direction.

Yang et al. fabricated free‐standing BP liquid‐crystalline films with different structural colors and investigated the effect of reactive mesogen and nonmesogenic monomers on BP self‐assembly and photopolymerization process.^[^ ^169^ ^]^ They found that the non‐mesogenic monomer automatically filled the disclination lines of the BP during the self‐assembly process, which was beneficial for the formation of free‐standing BP polymer films with well‐organized photonic nanostructures by photopolymerization. Moreover, dual‐wavelength lasing with low threshold and high quality was first achieved in BP materials, which was attributed to the large‐domain texture of the free‐standing BP films. This work provided a new and significant insight into the self‐assembly process of BPLCs for the preparation of free‐standing BP films and high‐performance optical devices. Yang et al. further investigated the photonic shape memory behavior of such BP liquid‐crystalline films (Figure 15c,d).^[^ ^170^ ^]^ Multiple blue‐shift colors were realized by shape memory programming under different mechanical pressures. The blue‐shift colors were attributable to a decrease in effective BP pitch along the viewing direction due to the compression deformation of the BP films. The compressed BP films recovered their original shapes and selective reflections upon heating to temperatures above their glass‐transition temperature. The efficient, facile, and low‐cost strategy for preparing free‐standing BP photonic materials will not only allow the fabrication of photonic shape memory polymers, but also enable their application in optical sensors and rewritable photonic papers.

**Figure 16:**
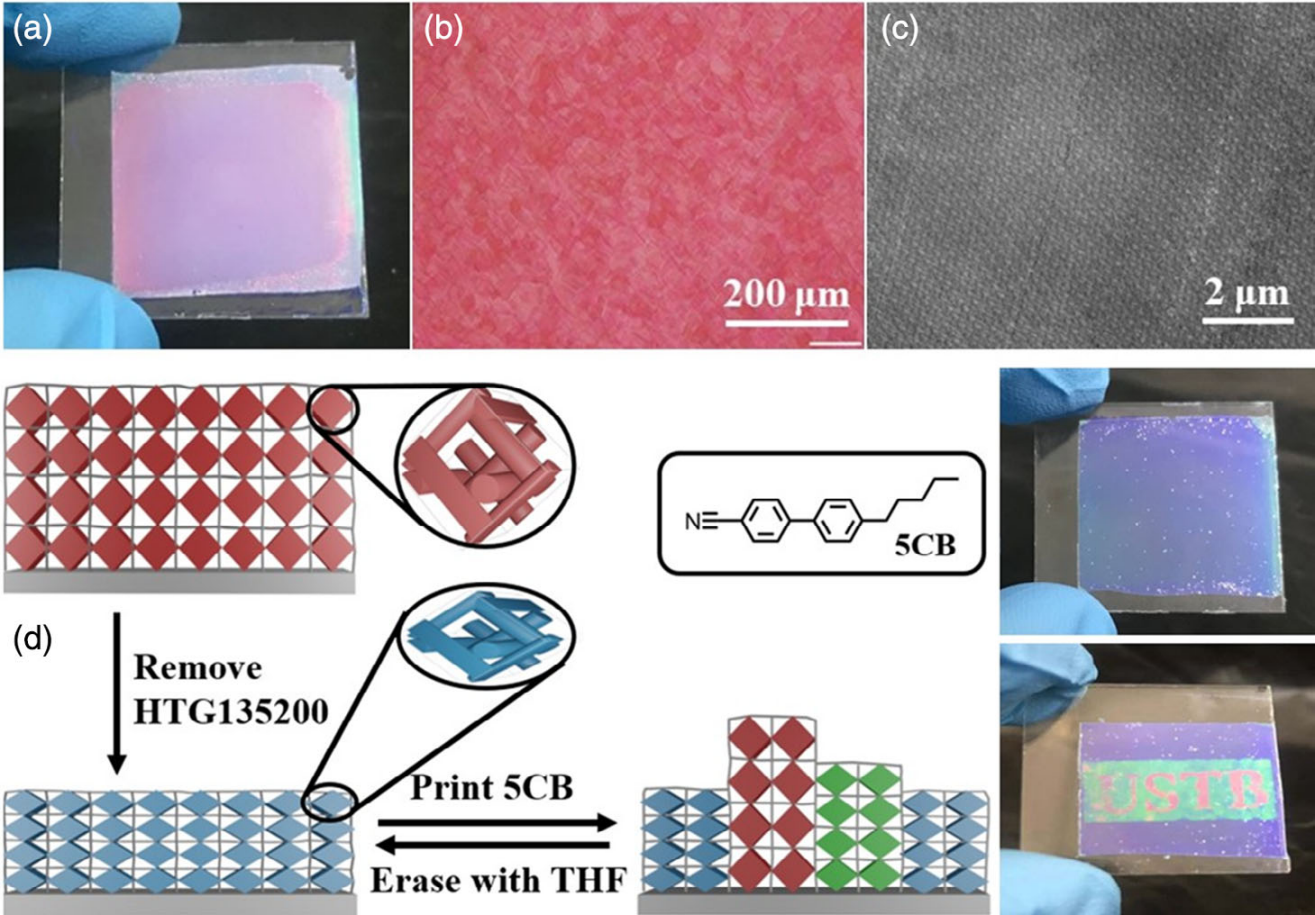
a) Photographs, b) POM image, and c) TEM image of the original BP polymer coating. d) Schematic of the working principle and photographs of multicolor pattern in the BP polymer coating. The variation in reflection colors is a result of the swelling/deswelling of the polymer network induced by the absorption/desorption of the liquid‐crystal‐ink, leading to a change in BP lattice in the film. a–d) Reproduced with permission.^[^ ^171^ ^]^ Copyright 2019, Royal Society of Chemistry.

Partially polymerized BP liquid‐crystalline films can be used to prepare multicolor photonic polymer coatings.^[^ ^171^ ^]^ As shown in **Figure** **16**, a photonic polymer coating with a uniform structural color was prepared from a partially polymerized BP network. Because of the well‐designed mesogen composition of the BP precursors, beautiful large‐area monodomain BP textures were obtained by direct self‐assembly without any alignment layers in the liquid‐crystal cells (Figure 16a,b). The self‐assembled nanostructures of the monodomain BP polymer coating were analyzed by transmission electron microscopy (TEM), which showed large‐area arrangement of alternating bright and dark contrasts due to the periodic orientation of the molecules (Figure 16c). The BP polymer coatings were covalently bonded to glass substrates, which were patterned using a liquid‐crystal‐ink to swell the network. The degree of swelling was controlled by modulating the voltage of the inkjet printer, determined the printed amount of liquid‐crystal‐ink. As a result, arbitrary multicolor patterns covering the entire visible spectrum could be printed, erased, and reprinted. As shown in Figure 16d, a University Science and Technology Beijing (USTB) pattern was printed and stored under ambient conditions for 30 days with no obvious change in the position of the selective reflections of the three colors. This indicates that the photonic patterns were extraordinarily stable under ambient conditions. These BP polymer coatings are promising materials for application as multicolor displays and rewritable photonic papers.

Kitzerow et al. first reported fully polymerized BP polymer films in which a 100 wt% reactive mesogen system was used to generate BP networks.^[^ ^172^ ^]^ These BP textures can be permanently stored by short UV light exposure; however, the BP films exhibited poor optical performance after photopolymerization. The light intensity was found to play a crucial role in the polymerization reaction. The use of low‐intensity light for photopolymerization in a chiral monomer system can result in the pitch gradient of the reflection band or even induce phase transition. In contrast, high‐intensity light expedites the photopolymerization process, which facilitates the retention of the chiral photonic structure after photopolymerization. Tanaka et al. applied high‐intensity UV light (≈400 mW cm^−2^) for rapid photopolymerization.^[^ ^173^ ^]^ The BP samples were polymerized rapidly so that the BP order could be preserved in the free‐standing films. Moreover, the DTC arrangements in BP I and BP II were analyzed by real‐space TEM imaging and compared. Fully polymerized BP polymer films exhibit self‐assembly similar to traditional low‐weight liquid crystals; however, photopolymerization leads to the in situ quenching of the superstructure, resulting in the formation of high‐quality films with well‐organized lattice orientations.

**Figure 17:**
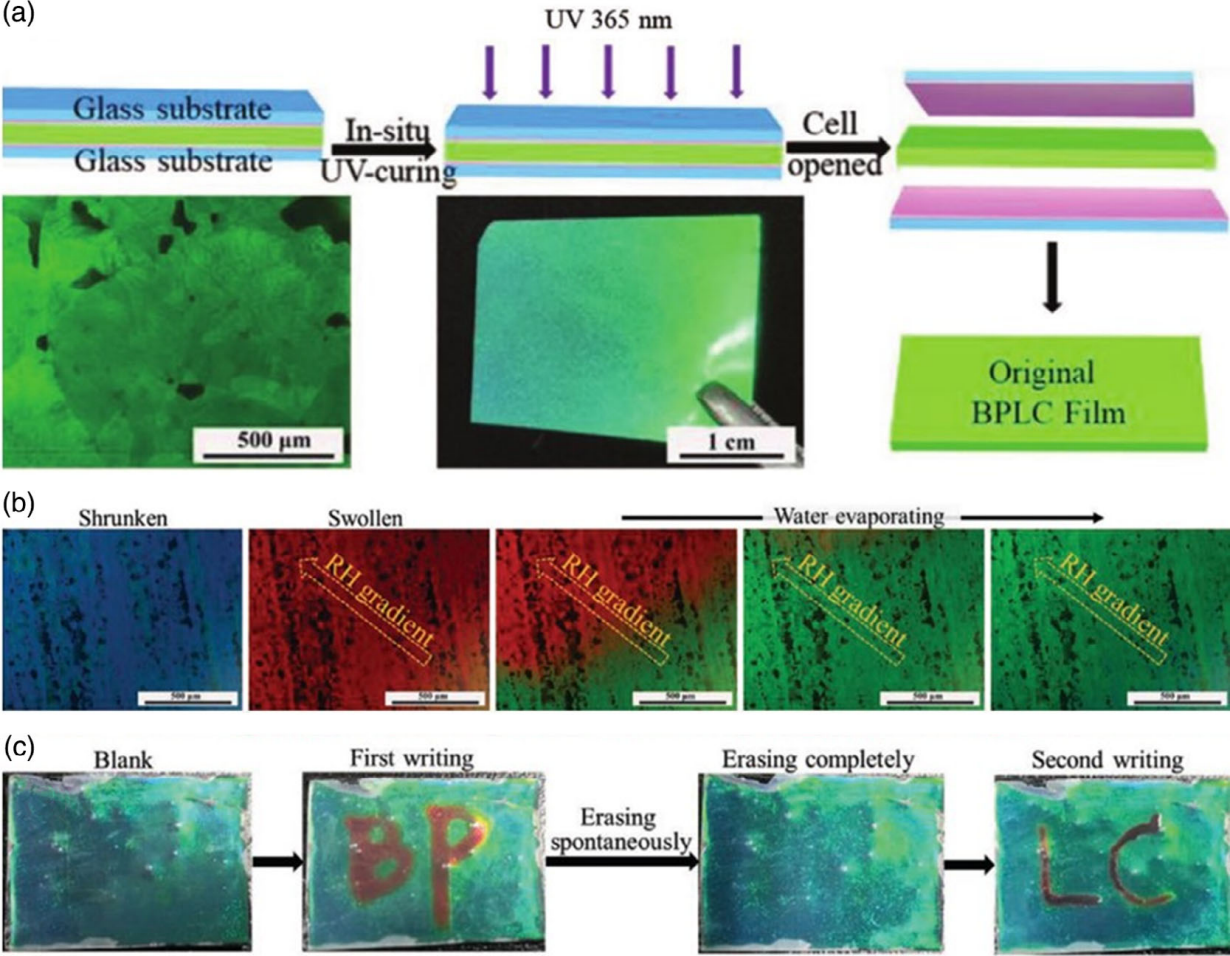
a) Schematic of the fabrication procedure of a free‐standing BP polymer film. b) POM images of the color changing with the relative humidity. c) Photographs of the writing and erasing show of the film. a–c) Reproduced with permission.^[^ ^174^ ^]^ Copyright 2020, Wiley‐VCH.

Hu et al. constructed a novel material system with a wide temperature range of BP films consisting of completely polymerizable monomers and a high concentration of H‐bonded units.^[^ ^174^ ^]^ After photopolymerization, the self‐assembled BP photonic nanostructures were successfully fixed, producing a humidity‐responsive free‐standing BP polymer film with reconfigurable, reprogrammable, and visually detectable optical properties (**Figure** **17**a). The change in the lattice scale of the self‐assembled 3D periodic nanostructures in the BP film resulted in the shifting of reflection color. The humidity‐responsive BP film was used in battery‐free multicolored displays with good recyclability as well as for humidity detection, anticounterfeiting, and information encryption (Figure 17b,c). Such free‐standing 3D photonic nanomaterials or other soft materials with extraordinary responsive properties have potential application in innovative devices and technologies.

Fully polymerized BP films with excellent optical and mechanical properties can be used in extreme environments such as extremely high or low temperatures, extreme pressure, and harsh solvents. Zhang et al. used a fully reactive mesogenic system to construct stretchable free‐standing BP polymer films.^[^ ^175^ ^]^ The mechanical reversible selective reflections of the BP film could be flexibly tuned within 135 nm and the mechanical tuning of laser emission in a wide spectral range could be further improved. In addition, the BP films exhibited high thermal and chemical stabilities; hence, their sharp photonic bandgaps in the entire visible range can be maintained even after thermal treatment (over 200 °C) and solvent treatment. The free‐standing BP film can serve as a platform for the development of functional and intelligent materials for application in displays, sensors, lasers, detection, optical devices, and microrobots.

## 6 Conclusion

We present a state‐of‐the‐art review of 3D chiral photonic nanostructures based on BPLCs and their potential applications. Liquid‐crystalline BP nanostructures are expected to self‐assemble into massive monodomain or monocrystalline architectures with narrow selective Bragg reflection bands through lattice orientation modulation. Because of their remarkable 3D photonic properties arising from self‐organization and ultrasensitivity to various external stimuli, these BP nanostructures can selectively and efficiently change reflective colors in a controllable or even programmable manner, which enables their promising applications in a variety of advanced multifunctional photonic devices. Furthermore, in recently years, free‐standing liquid‐crystalline BP polymer films have been developed, which not only overcome the limitations of liquid‐crystal cells, but also exhibit superior optical and mechanical performance. The distinctive and inherent chiral, optical, and flexible properties of these free‐standing 3D photonic films will undoubtedly endow the resultant materials and photonic devices with exceptional functions.

Despite significant advancements, the development of BP‐based photonic nanostructured materials is still in its nascent stage. To realize the full potential of BPLCs with 3D chiral photonic nanostructures in wide commercial applications, many critical issues and basic challenges need to be addressed. It is still a formidable task to develop commercially viable low‐molecular‐weight BPLC hosts that can meet different application requirements such as stable 3D nanostructures over a wide temperature range from 80.0 to −30.0 °C. According to the Landau‐de Gennes theory,^[^ ^80^ ^]^ stable BPLCs could be achieved by effectively filling the defect cores and reducing the total free energy of 3D chiral nanostructures. The underlying mechanism and chiral nanostructures should be experimentally elucidated with the help of emerging techniques such as resonant soft X‐ray scattering (RSoXS)^[^ ^176^ ^]^ and cryogenic transmission electron microscopy (cryo‐TEM).^[^ ^177^ ^]^ In 2017, RSoXS was first applied for characterizing 3D nanostructures of BPLCs,^[^ ^178^ ^]^ and the transformation between BPI and BPII phases has been recently revealed by RSoXS study of directed single domain.^[^ ^133^ ^]^ BPLCs with many different crystal symmetries such as quasi‐2D skyrmion lattices and beyond have been theoretically predicted, however, the experimental fabrication of these photonic nanostructures and controllable growth of micrometer‐sized large single crystals are still challenging.^[^ 179, 180 ^]^ As 3D chiral soft nanostructures, the significance of chirality in BPLCs and corresponding chirality‐related optical devices and other applications are still less explored arena with exciting future. Taking advantages of the unique 3D nanostructures, BP‐based soft materials could exhibit faster responsivity and higher sensitivity than traditional cholesteric materials in some cases. For example, ultrafast submillisecond response speed can be expected in information display and other optical devices under electric field when the BPLCs with low viscosity and high dielectric constant are applied.^[^ ^96^ ^]^ Recently, BP‐based polymeric films were found to show an optical response to toluene vapor that was sixfold faster in sensitivity than the polymerized nematic or cholesteric phases and with a limit of detection.^[^ ^165^ ^]^ Free‐standing BP film with 3D chiral nanostructures that respond to diverse external stimuli as well as mimic natural coloration and functions could serve as a platform for the development of functional and intelligent materials toward diverse applications, however, scalable‐manufacture of these flexible optical films remains scarce.^[^ 181, 182, 183 ^]^ It is worth noting that BPLCs are significantly promising as a template for the self‐assembly of nanoparticles or polymers due to their inherent 3D disclination networks.^[^ 112, 184, 185, 186 ^]^ This is expected to open new avenues for the development of 3D nanostructures with immense potential for photonic and plasmonic applications. Last but not the least, the architectures of BPLCs with left‐handed or right‐handed circular dielectric spirals along the three orthogonal spatial axes can inspire the design of artificial 3D chiral photonic objects, which are currently in great demand in nanotechnology and microelectronics industry.^[^ 187, 188, 189, 190 ^]^ Various strategies have been proposed to fabricate 3D chiral nanostructures (single and periodic) and metamaterials with the precise control of the nanoscale geometry, such as focused ion beam lithography,^[^ 191, 192, 193, 194 ^]^ glancing angle deposition,^[^ 195, 196 ^]^ 3D nanoimprint lithography,^[^ 197, 198 ^]^ and direct laser writing.^[^ 199, 200, 201 ^]^ These studies pave the way for the creation of novel and advanced nanophotonic devices for a wide range of applications such as polarization control, integration in photonic circuits, and subwavelength imaging. BP‐based photonic materials will undoubtedly continue to be a hot research subject in the fields of liquid crystals and nanoscience, which may lead to the development of programmable and reconfigurable materials with more fascinating functions and unlimited possibilities. The dynamic and exciting progresses in the past decades have provided a robust foundation for understanding the unique properties of 3D chiral photonic materials. Continuous pioneering research on such promising areas is expected to not only extend our knowledge of soft photonics, but also accelerate their multitudinous application as advanced intelligent materials.

## Conflict of Interest

The authors declare no conflict of interest.
